# Single Nucleotide Polymorphisms, Structural Variants, and Short Tandem Repeats Capture Distinct Signals of Adaptive Divergence in the Atlantic Puffin

**DOI:** 10.1093/gbe/evaf162

**Published:** 2025-08-19

**Authors:** Oliver Kersten, Bastiaan Star, Tycho Anker-Nilssen, Hallvard Strøm, Kjetill S Jakobsen, Sanne Boessenkool

**Affiliations:** Centre for Ecological and Evolutionary Synthesis (CEES), Department of Biosciences, University of Oslo, Oslo, Norway; Centre for Ecological and Evolutionary Synthesis (CEES), Department of Biosciences, University of Oslo, Oslo, Norway; Norwegian Institute for Nature Research (NINA), Trondheim, Norway; Fram Centre, Norwegian Polar Institute, Tromsø, Norway; Centre for Ecological and Evolutionary Synthesis (CEES), Department of Biosciences, University of Oslo, Oslo, Norway; Centre for Ecological and Evolutionary Synthesis (CEES), Department of Biosciences, University of Oslo, Oslo, Norway

**Keywords:** structural variation, short tandem repeats, single nucleotide polymorphisms, adaptation, conservation genetics

## Abstract

The Arctic has been the scene for (re)colonization, diversification, and adaptation of boreal and Arctic fauna. As anthropogenic warming of the Arctic environment increases the extinction risk for peripheral populations, understanding patterns of local adaptation is imperative. The Atlantic puffin (*Fratercula arctica*) comprises multiple genetically and morphologically distinct populations with an Arctic-boreal distribution. Yet, patterns of adaptation between these populations remain poorly understood. Here, we investigate potential adaptive divergence between High Arctic (*F. a. naumanni*) and boreal (*F. a. arctica*) puffin subspecies using whole-genome sequence data. We analyze different types of intraspecific DNA variation, including single nucleotide polymorphisms (SNPs), structural variants (SVs) and short tandem repeats (STRs). Patterns of elevated levels of genetic divergence vary across these types, with STRs uncovering the largest unique proportion (47.2%) of genomic outlier loci. Notably, 94.5% of all outlier genes are exclusive to one type of variation and several such genes are linked to phenotypic differences observed between these subspecies, including body size, skeletal development, adipose tissue accumulation and the sensory system. Our observations indicate that *F. a. naumanni* harbors unique genetic diversity within puffins suggesting adaptation to its Arctic environment. Importantly, we show that SNPs, SVs, and STRs capture distinct signals of adaptive divergence, underscoring the importance of integrating multiple genomic markers to fully understand the complexity of local adaptation. These results offer a broader perspective on genomic patterns of adaptive divergence in Arctic fauna and can inform conservation strategies aimed at preserving genetic diversity in the Atlantic puffin.

Significance In the face of exacerbated ecological impacts on Arctic fauna due to climate change, understanding the genetic basis of adaptation in Arctic species is crucial, yet remains poorly understood. Here we analyze the genomes of a boreal and High Arctic populations of the iconic Atlantic puffin. We find that the assessment of genetic differentiation and adaptive divergence requires the integration of several types of DNA variation (single nucleotide polymorphisms, structural variants, and short tandem repeats), and we identify several genomic regions potentially playing a role in the adaptation of Atlantic puffins to the Arctic environment. These insights enhance our understanding of the complex spectrum of adaptive genetic diversity in Arctic species and the importance of integrating multiple genomic markers.

## Introduction

The Arctic is a unique ecosystem that hosts a diverse array of locally adapted species (Blix 2016; Yudin et al. 2017; Castruita et al. 2020; Colella et al. 2020). However, this biodiversity is under the immediate threat from climate change and other environmental stressors, with impacts that are exacerbated in the Arctic (eg Serreze and Barry 2011; Croxall et al. 2012; Colella et al. 2020; CAFF 2021; Stroeve et al. 2025). To understand how Arctic species and populations may cope with rapid environmental shifts, it is essential to investigate whether adaptive genomic variation underlies observed phenotypic and ecological differences. Genomic studies of Arctic adaptation often pinpoint specific genomic regions or genes linked to thermal adaptation, encompassing traits such as adipose tissue development, metabolism, cardiac gene regulation, pigmentation, and other energetic pathways. Nonetheless, the majority of these findings rely solely on the analysis of single nucleotide polymorphisms (SNPs) or transcriptomics (Kumar et al. 2015; Yudin et al. 2017; Castruita et al. 2020; Colella et al. 2020). Similarly, most population genomic analyses of local adaptation across other ecosystems have focused on outlier loci based on SNPs only by linking differences in allele frequencies along environmental gradients or between populations to functional or phenotypic differences (Luikart et al. 2019; Hohenlohe et al. 2021). However, while SNPs have provided valuable insights, they may capture only a subset of the variation underpinning adaptation and divergence (Mérot et al. 2020; Wold et al. 2021; Campagna and Toews 2022). In fact, recent research highlights the importance of integrating additional forms of genetic variation, such as structural variants (SVs such as inversions, copy number variations, and rearrangements) and short tandem repeats (STRs), which influence gene regulation, expression, and function (eg Dorant et al. 2020; Jakubosky et al. 2020; Weissensteiner et al. 2020; Cayuela et al. 2021; Reinar et al. 2021, 2023, 2024; Wold et al. 2021; Campagna and Toews 2022; Mérot et al. 2023; King 2025). Comparative studies across taxa—including eg American lobster (*Homarus americanus*), humans (*Homo sapiens*), and European starlings (*Sturnus vulgaris*)—demonstrate that outlier loci often differ between variant types and rarely overlap, emphasizing their complementary roles in capturing adaptive variation (Dorant et al. 2020; Jakubosky et al. 2020; Stuart et al. 2023). Distinct differences in mutation rate between variant types contribute to this complementarity. Indeed, STRs mutate at rates orders of magnitude higher than SNPs (Fan and Chu 2007; Willems et al. 2014), potentially making them well-suited candidates to drive rapid evolution or divergence (Reinar et al. 2024; Zhang et al. 2024; King 2025). Nevertheless, few studies have jointly examined SNPs, STRs, and SVs at the population level, and almost none have explored their contributions to adaptive divergence in nonmodel species (Mortazavi et al. 2022; Zhang et al. 2024).

While a more complete representation of DNA variation beyond SNPs is needed to disentangle genomic patterns of adaptation within populations and their associations with phenotypic differences (Wold et al. 2021), detecting SVs and STRs presents distinct methodological challenges. For instance, detection rates vary among different SV types due to platform-specific technical biases (Ho et al. 2020). Moreover, short-read sequencing technologies struggle to accurately detect large SVs, particularly when variants exceed the read length or occur in long stretches of repetitive regions or when reference genomes are incomplete and discontinuous (Hall and Quinlan 2012; Sedlazeck et al. 2018; Chaisson et al. 2019; Mahmoud et al. 2019; Zhou et al. 2019). Despite these limitations, short reads remain a practical and widely used tool for SV discovery in population genomics even if analyses and results still require careful curation and interpretation (Mahmoud et al. 2019; Reinar et al. 2021, 2023; but see Bertolotti et al. 2020).

Here, we present a whole-genome analysis of divergence between two subspecies of the Atlantic puffin (*Fratercula arctica*), an iconic North Atlantic seabird species that is known for its brightly colored bill and distinctive black and white plumage (Harris and Wanless 2011). Currently, the Atlantic puffin is endangered in Europe due to decades of poor breeding success and subsequent population declines in the central part of its breeding range (BirdLife International 2017; Layton-Matthews et al. 2023) and comprises four distinct genetic clusters, partially corresponding to the three traditionally recognized subspecies: *F. a. naumanni, F. a. arctica*, and *F. a. grabae* (hereinafter *naumanni*, *arctica*, *grabae*; Harris and Wanless 2011; Kersten et al. 2021; Leigh et al. 2023). These subspecies differ in some phenotypic and ecological traits that vary along a latitudinal gradient, but are most pronounced in *naumanni*, the subspecies that is restricted to the Arctic (Salomonsen 1944; Pethon 1967; Harris and Wanless 2011; Burnham et al. 2020; Kersten et al. 2021; Leigh et al. 2023). However, as with many species inhabiting the remote Arctic, detailed phenotypic data of puffins is limited. Among the traits that are well characterized, body and bill size stand out with *naumanni* being significantly larger than the other subspecies. Such size-related traits are known to be highly polygenic and influenced by variation across many genes. Additionally, *naumanni* exhibits pronounced differences in ecology, migration timing, and diet (Barrett et al. 2002; Harris and Wanless 2011; Underwood 2019; Anker-Nilssen and Bangjord 2021; Burnham et al. 2021). Genomic reconstructions suggest that *naumanni* and *arctica* initially diverged during climatic fluctuations in the Pleistocene (Kersten et al. 2023). Recent research has also provided evidence for recent gene flow and subsequent hybridization between the two subspecies due to recent environmental change leading to the formation of a hybrid population in the High Arctic (Kersten et al. 2021, 2023). Despite previous research on the demographic history of Atlantic puffin subspecies, we still lack a comprehensive understanding of their genomic differentiation and adaptive divergence. Furthermore, we currently possess no understanding of differences in rates of gene flow, recombination, and mutation between the subspecies, which may influence the extent and nature of their genomic divergence.

**Fig. 1.**
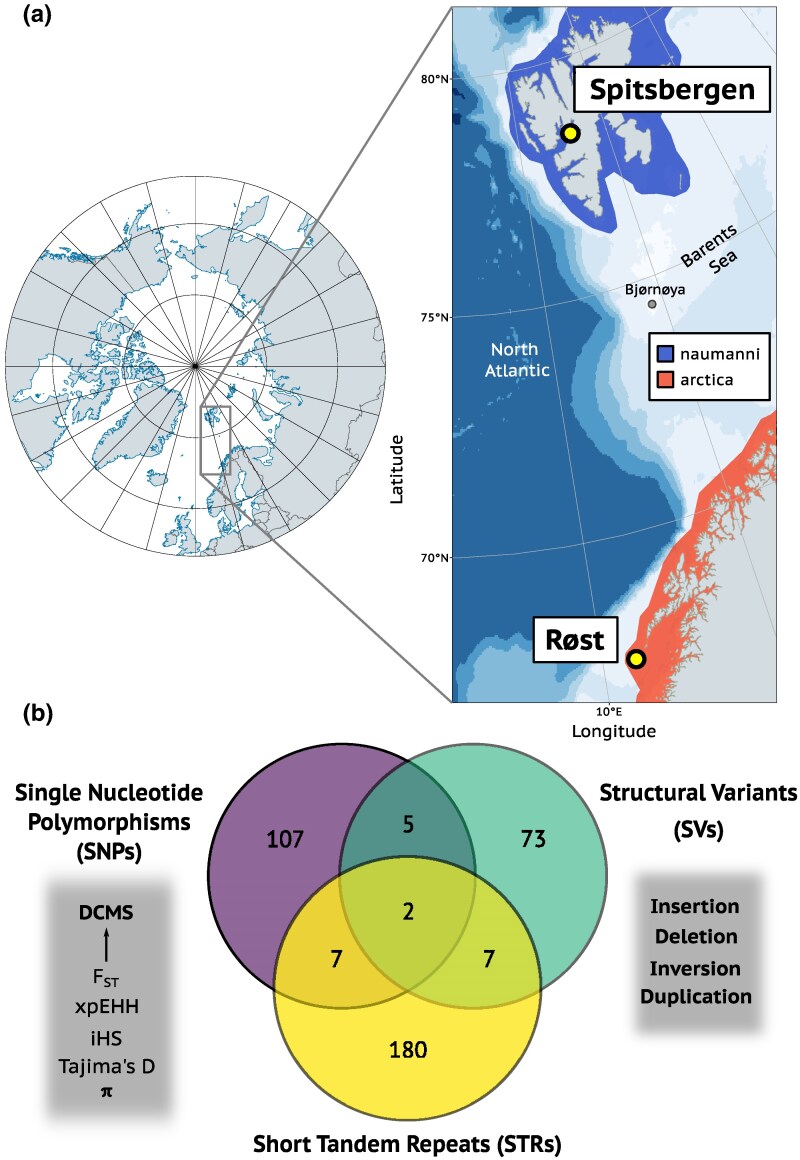
Outlier loci and gene detection between two Atlantic puffin subspecies. a) Map displaying the study area with the three colonies from which sequencing data was analyzed. Spitsbergen and Røst are representative colonies of the subspecies *naumanni* and *arctica*, respectively. Subspecies distributions are shown in blue and red, and the continuous color scale from blue to white indicates depth of ocean basins. b) Venn diagram showing the overlap of genes falling within 8 kb of outlier loci detected by genome scans of three different types of DNA variation (SNPs, STRs, and SVs). For eg five separate genes were found in close proximity to both SNP and SV outlier loci. The DCMS combines five different SNP-based genome statistics commonly used for selection scans, which increases detection power.

Here, we analyze previously published whole-genome sequencing data from 18 Atlantic puffins (Kersten et al. 2023) using advanced bioinformatics workflows to detect outlier regions associated with SNPs, SVs, and STRs (Fig. 1). This study is among the first to date to incorporate these three types of genetic variation in a comparative framework. Our results identify numerous outlier loci in close proximity to genes potentially associated with phenotypic differences between subspecies, including traits such as body size, skeletal development, and fat accumulation. Most outliers were unique to a single type of genomic variation, underscoring the importance of multivariant approaches to comprehensively capture genomic signatures of divergence and adaptation. STRs contributed disproportionately to intersubspecific differentiation and showed substantial enrichment near genes involved in polar adaptation. Collectively, this study offers novel insights into the genomic basis of divergence that may underlie local adaptation in a highly mobile Arctic seabird. The results also highlight the value of integrating multiple types of DNA variation in the analyses of adaptive divergence. By uncovering distinct genomic regions potentially linked to phenotypic and ecological differentiation, our findings contribute to identifying evolutionarily significant units (ESUs)—populations that are genetically and ecologically distinct and therefore warrant separate conservation management (Funk et al. 2012; Hohenlohe et al. 2021). Recognizing ESUs is critical for preserving adaptive potential and evolutionary resilience, especially in rapidly changing environments like the Arctic (Funk et al. 2012; Supple and Shapiro 2018; Hohenlohe et al. 2021; Wold et al. 2021). Together, our findings provide a starting point for future research and inform conservation efforts by refining our understanding of subspecies divergence in the Atlantic puffin.

## Results

This study utilizes genomic data from 18 Atlantic puffins to investigate genetic variation between the subspecies *Fratercula arctica naumanni* and *F. a. arctica*. Employing whole-genome sequencing to a genome-wide average depth of coverage of 21.6×, the dataset encompasses SNPs, SVs, and STRs to identify outlier loci. These outliers were further analyzed in relation to protein-coding genes and gene ontology (GO) to pinpoint candidate genes associated with phenotypic and habitat differences, which may underlie adaptive divergence between the subspecies.

### SNP-Based Genome Selection Scans

**Fig. 2.**
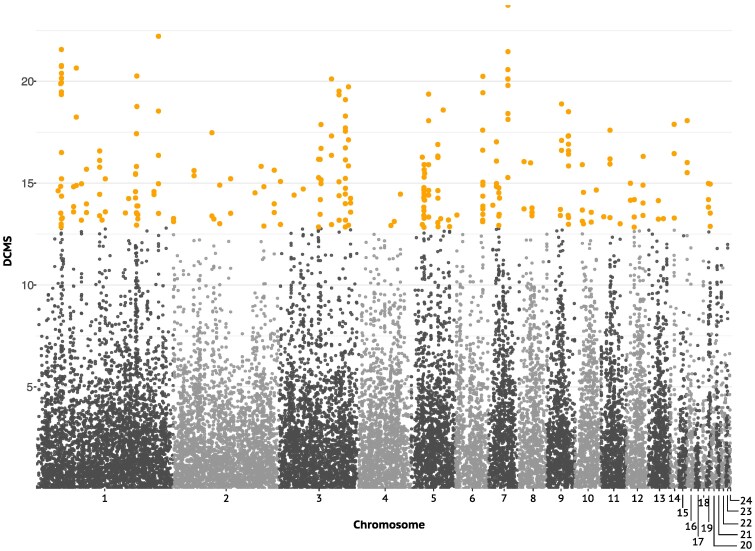
Autosomal distribution of the DCMS between two Atlantic puffin subspecies based on SNPs. The DCMS combines five different SNP-based genome statistics commonly used for selection scans (iHS, xpEHH, Tajima's D, π, and *F*_ST_), which increases detection power. Values were calculated in 50 kb sliding windows (25 kb slide). Significant outliers (*q* < 0.05) are highlighted in yellow. Chromosome boundaries are highlighted by an alternating light and dark gray pattern.

After correction for multiple testing, the genome-wide decorrelated composite of multiple signals (DCMS)—which combines information on allele frequencies, phased haplotypes, and genetic differentiation—identified 255 outlier windows (50 + kb windows) out of a total of 43,638 windows that were distributed across all autosomes (Fig. 2; Fig. S1 and S2). Utilizing the DCMS statistic, rather than solely relying on *F*_ST_, has an improved sensitivity to detect outliers more effectively. This sensitivity is evident from the lack of higher *F*_ST_ values observed within DCMS outlier windows, compared to nonoutlier windows that are proximate to introns, coding sequences (CDS), and intergenic areas (Fig. S3). The 255 outlier windows were merged into 104 nonadjacent regions, of which 60 were supported by at least two overlapping outlier windows. After confirming that the distribution of distances from outlier and nonoutlier DCMS windows to the nearest CDS showed no substantial differences (Fig. S4), a distance cutoff of half the average distance between two protein-coding genes (8 kb) in the Atlantic puffin genome annotation was chosen. Of the 60 regions, a total of 49 (81.67%) were located within 8 kb of 121 different protein-coding genes (Fig. 1; Fig. S5). Despite a large fraction of outlier windows being in proximity to protein-coding genes, these windows do not have a significantly higher or lower occurrence within 8,000 bp upstream and downstream of genes as expected by chance given their length (permutation test, 1,000 permutations, *P* > 0.05).

We investigated genome-wide and within outlier windows for patterns of correlations between *F*_ST_, *d_XY_* and π, and assessed differences in these parameters between outlier and nonoutlier windows. Nucleotide diversity (π) was strongly positively correlated between subspecies (Pearson correlation coefficient), both across the whole genome (*r*[43,754] = 0.96, *P* < 2.2 × 10^−16^) and in outlier windows (*r*[253] = 0.90, *P* < 2.2 × 10^−16^) (Fig. S6). Similarly, the relationship between *d_XY_* and mean π across subspecies was significant and strongly positive both genome-wide (*r*[43,754] = 0.99, *P* < 2.2 × 10^−16^) and in outliers (*r*[253] = 0.98, *P* < 2.2 × 10^−16^) (Fig. S6). There was also a weak, yet significant, positive relationship between *F*_ST_ and *d_XY_*, across the entire genome (*r*[43,754] = 0.011, *P* = 0.02) and in outlier windows (*r*[253] = 0.16, *P* = 0.009), while the correlation between *F*_ST_ and the mean π across subspecies was significantly negative genome-wide (*r*[43,754] = −0.096, *P* < 2.2 × 10^−16^) and nonsignificant in outlier windows (*r*[253] = −0.019, *P* = 0.76). Additionally, *F*_ST_ appeared to be elevated in regions with higher *d_XY_* relative to the mean π and where both subspecies had low π (Fig. S6). Assessing differences between outlier and nonoutlier windows, *F*_ST_ was significantly higher (*W* = 365,860, *P* < 2.2 × 10^−16^) and *d_XY_* significantly lower (*W* = 7,250,856, *P* < 2.2 × 10^−16^) in outlier versus nonoutlier windows (Fig. S7). While being more pronounced in naumanni, nucleotide diversity was significantly lower in outlier windows of both subspecies (*naumanni*: *W* = 9,361,890, *P* < 2.2 × 10^−16^; *arctica*: *W* = 7,226,207, *P* < 2.2 × 10^−16^; Fig. S7).

### Structural Variant Analyses

**Fig. 3.**
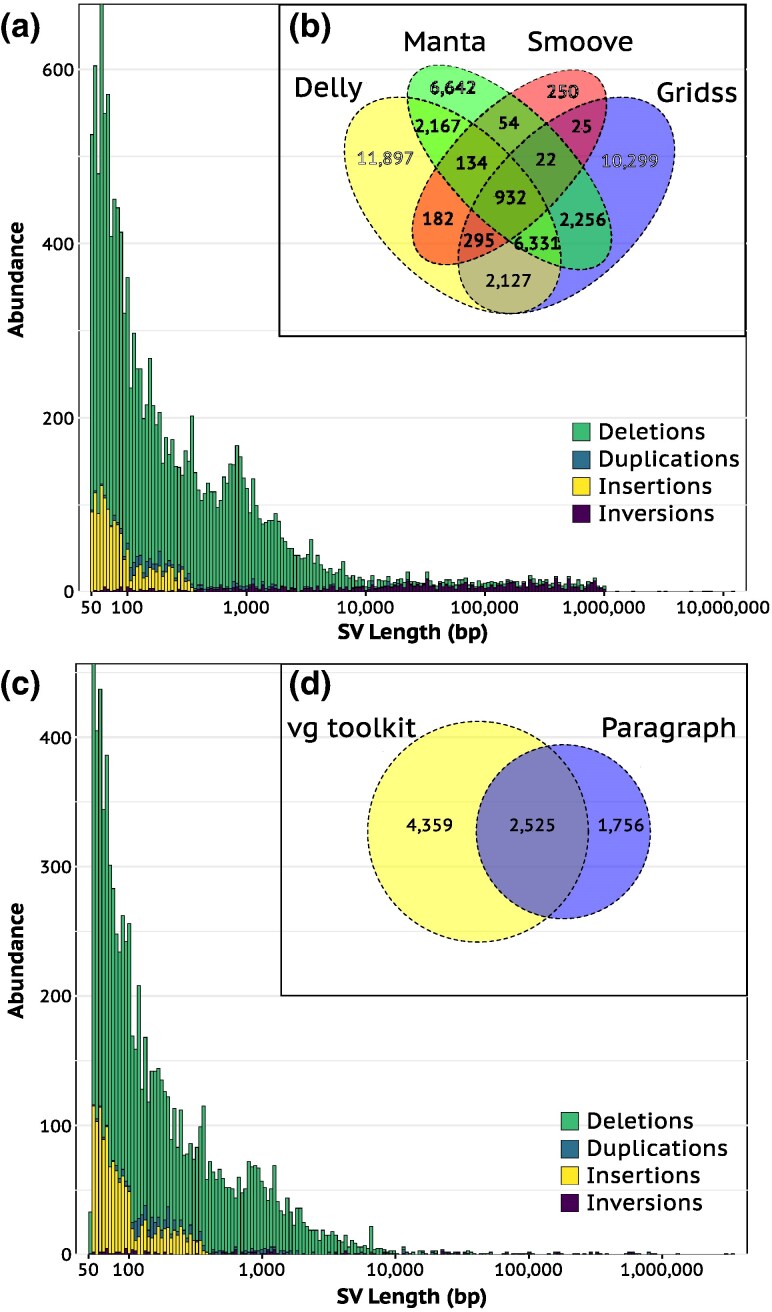
Length and identity of structural variants detected and genotyped among Atlantic puffin genomes using short-read sequencing data. a) The number and length of four different types of SVs that were identified by at least two of the four used detection programs (Delly, Manta, Smoove, Gridss). b) Venn diagram displaying the overlap of detected SVs among the used detection programs. c) The number and length of four different types of structural variants (SVs) that were successfully genotyped by either of the two programs, vg toolkit and Paragraph. d) Venn diagram displaying the overlap of genotyped SVs among vg toolkit and Paragraph. STRs < 100 bp were detected and called by a different pipeline.

The combination of four different programs (Delly, Smoove, Manta, and Gridss; see Materials and Methods) for structural variant identification revealed 43,613 SVs across puffin genomes of the Spitsbergen, Røst, and Bjørnøya colonies, of which 14,525 (33.3%) were detected by at least two programs and kept for further analyses (Fig. 3; Fig. S8). The vast majority of these retained SVs were deletions (82.68%). The programs varied greatly in their ability to detect the different SV types and the applied filters affect each program differently (Fig. S8). For instance, Smoove was not able to detect insertions (Layer et al. 2014) and duplications found by Smoove were removed by stringent filtering (Fig. S8). After assigning genotypes and further filtering (Supplementary File 1), a total of 8,640 (59.48%) of the 14,525 SVs were retained. Similar to the SV detection tools, SV genotype callers varied in their ability to call genotypes for the different SV types (Fig. S9), as observed in previous studies (eg Chen et al. 2019; Hickey et al. 2020). Specifically, there was no overlap in the called insertions, and while Paragraph was able to call genotypes for almost all inversions and duplications that were called by the vg toolkit, it only called one-third of the deletions that were called by the vg toolkit (Fig. S9). Calling and filtering genotypes of the SVs also shifted the highly skewed distribution of SV sizes further toward small SVs below 500 bp and disproportionately removed long inversions (Fig. 3). As a result, the calling and filtering process also removed the apparent artifact that SVs had a sharp drop in abundance at 1 Mb (Fig. 3a), which is unsurprising since SVs over 1 Mb are likely not confidently detected using short-read data (Sedlazeck et al. 2018). Despite removing 80.19% of initially detected SVs, the final set was able to reconstruct the genomic population structure previously identified using SNPs with Spitsbergen and Røst being on either side of PC1 (Fig. S10). Yet, there were no fixed differences between the two subspecies, ie none of the SVs that were called in all individuals were fixed for the alternate allele in *naumanni* and fixed for the reference allele in *arctica*. Finally, the quality of the SV data was deemed robust, as evidenced by the relationships between sequencing depth and SV length, as well as between *F*_ST_ and missingness (Figs. S11 and S12).

After calculating *F*_ST_ values for each of the 8,640 SVs, fitting them to a normal distribution and correcting for multiple testing (FDR < 0.05), 144 SVs were identified as outliers (*q* < 0.05). Outlier SVs were composed of 103 deletions, 10 duplications, 29 insertions, and two inversions. Of those, 83 were within 8 kb of 87 different protein-coding genes (Fig. 1; Fig. S5), with two duplications and one deletion situated within CDS of the CACNA2D1 (Calcium Voltage-Gated Channel Auxiliary Subunit Alpha2 Delta 1), PRR14L (Proline Rich 14 Like), and KLHL3 (Kelch Like Family Member 3) genes. Outlier SVs generally did not have a significantly higher or lower occurrence within 8,000 bp upstream and downstream of genes as expected by chance (permutation test, 1,000 permutations, *P* > 0.05).

### STR Analyses

**Fig. 4.**
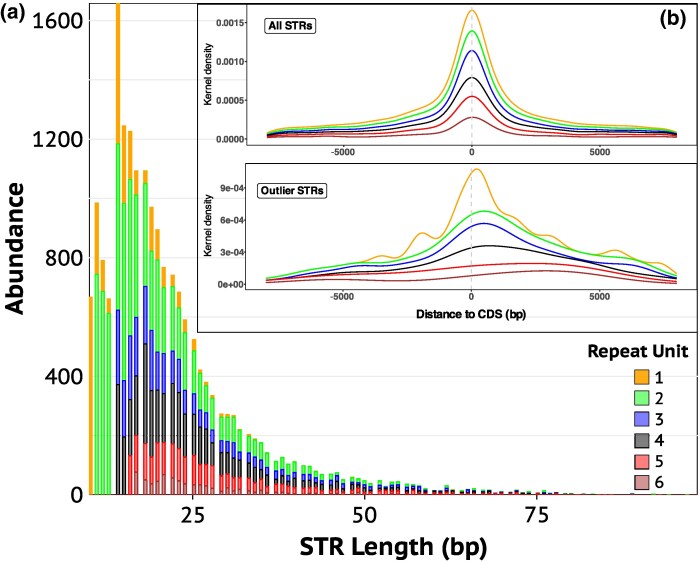
Description of STRs genotyped among Atlantic puffin genomes using short-read sequencing data. a) The number and length of STRs with different periods that were identified and genotyped by our bioinformatics pipeline. b) Densities of genome-wide and outlier STRs in relation to distance to CDSs (transcription start) within protein-coding genes. Different line colors denote different STR unit sizes (see legend). Kernel density is a nonparametric probability density estimation of the abundance of STRs in close proximity around the known STRs.

To assess the relationship between STRs, genes, and subspecies differentiation, STRs were identified and genotyped along the genomes of puffins from Spitsbergen, Bjørnøya, and Røst. The initial scan for STRs using HipSTR revealed 374,612 STRs, which were subsequently filtered to the final set of 19,260 STR sites (Fig. 4). The majority of STRs consisted of nucleotide homopolymer and dimer STR motifs and the overall length distribution was highly skewed for each motif type (unit sizes: 1, 2, 3, 4, 5, and 6) toward a length of <25 bp (Fig. 4). About 60% of the STRs were located within 8 kb of protein-coding genes and a PCA based on all STR genotypes recovered the same genomic population structure as observed with SNPs and SVs (Fig. S10).

Fitting Jost's *D* values of each STR to a normal distribution and correcting for multiple testing (FDR < 0.05) identified 326 outlier STRs (*q* < 0.05). 178 of those were situated within 8 kb of 196 unique protein-coding genes (Fig. 1; Fig. S5), with the large majority (78.09%) being in introns and none in CDS. While most of the outlier STRs were <30 bp long and comprised of homopolymer and dimer motifs, the trimer, tetramer, pentamer, and hexamer motifs were relatively more abundant in the longer outlier STR fractions of up to 80 bp (Fig. S13). Independent of unit size, both genome-wide STRs as well as outlier STRs were substantially enriched close to the start and end of CDS within protein-coding genes (Fig. 4).

### Outlier Genes and GO Analyses

Assessing data quality and providing context for interpreting genome-wide patterns, density plots for filtered SNPs, STRs, and SVs across each chromosome revealed no indications of technical artifacts or anomalous regions, supporting the robustness of the dataset and identification of outlier windows (Fig. S14). Across each of the three types of genomic variation, no fixed synonymous or nonsynonymous differences were identified between the two Atlantic puffin subspecies (Table S1). Combining protein-coding genes located within 8 kb of outlier DCMS windows, SVs and STRs (designated as “outlier genes”) resulted in a final set of 381 unique outlier genes (Fig. 1). GO analyses with chicken and human reference databases resulted in 12, 9, and 83 significant, unique biological pathways using outlier genes identified in the SNP, SV, and STR, respectively (Supplementary Data File 1). Pooling all outlier genes (*N* = 381) detected 176 biological gene pathways with evidence of enrichment (Supplementary Data File 1). These included, among others, positive regulation of chondrocyte differentiation, olfactory bulb development, and growth hormone secretion (Table S2). In addition, outlier genes were cross-referenced against a manually compiled list of 2,275 genes associated with morphology, cold adaptation, skeletal processes or tissue adaptation. Merging results of the GO and cross-reference analyses led to the identification of 40 outlier genes that are of specific interest, due to their putative role in the observed phenotypic differences between the subspecies *naumanni* and *arctica* (Table 1). Most of these genes are associated with changes in the skeleton and body size, and outliers close to these genes were largely not overlapping between the various types of polymorphisms (SNP, SV, or STR; Table 1), with STR outliers being in close proximity to a substantially larger proportion of genes of interest as DCMS outliers (Table 1). However, all three types of DNA variation comprised outliers in the *MID1* (*Midline 1*) and *PTPRK* (*Protein Tyrosine Phosphatase Receptor Type K*) genes (Fig. 1; Table 1). *PTPRK* encodes a protein of the protein tyrosine phosphatase family. These proteins are known to be signaling molecules that regulate a variety of cellular processes including cell growth, differentiation, mitotic cycle, and oncogenic transformation (Stelzer et al. 2016). As such, *PTPRK* could have several functions, but has notably been linked to changes in bill morphology in finches and sparrows (Lawson and Petren 2017; Gamboa et al. 2022). *MID1* encodes a protein that is likely involved in the formation of multiprotein structures acting as anchor points to microtubules (Stelzer et al. 2016), and has been shown to play a role in craniofacial processes as well as eye development in humans, chickens, mice and frogs (Baldini et al. 2020). Also, both STR and SV outliers were found in proximity to *CSMD3* (*CUB And Sushi Multiple Domains 3*), a gene involved in regulation of dendrite development and associated with differences in body size and cold adaptation in mammals (Yudin et al. 2017; Ghoreishifar et al. 2020). More than one quarter (12 out of 40) genes of interest, including *MID1,* have previously been associated with vision and olfactory system development (GO results, Table 1). Four of those 12 genes linked with vision and olfactory systems (*EPHA4*—*EPH Receptor A4*, *EYS*—*Eyes Shut Homolog*, *HS6ST1*—*Heparan Sulfate 6-O-Sulfotransferase 1*, *TOX*—*Thymocyte Selection Associated High Mobility Group Box*), together with *CABIN1* (*Calcineurin Binding Protein 1*) and *PCDH11X* (*Protocadherin 11 X-Linked*), harbored 12 of the 23 (11 in intergenic regions) fixed SNPs, where all Røst individuals were homozygous for the reference allele and all Spitsbergen individuals were homozygous for the alternate allele. These 12 SNPs were all located in introns (Table 1; Table S1).

**Table 1:** Genes potentially associated with local adaptation and phenotypic differences between two Atlantic puffin subspecies

Category	Gene name	Method	Gene ontology
**Skeleton, ossification, bone development, and body size**	ABI1	STR	Y
	ADK	STR	Y
	BMPR1B	STR	Y
	CSMD3	SV, STR	Y
	EP300	DCMS	Y
	GBX1	DCMS	Y
	GHSR	STR	Y
	GTF3A	STR	N
	HIVEP1	STR	Y
	INTU	STR	Y
	KL	SV	Y
	LRP3	DCMS	Y
	MID1	SV, STR, DCMS	Y
	NRP2	STR	Y
	PDCD4	DCMS	Y
	PPM1F	STR	Y
	PTPRC	STR	Y
	PTPRK	SV, STR, DCMS	Y
	RAF1	STR	Y
	RUNX2	STR	Y
	SHOX	STR	Y
	SLC33A1	DCMS	Y
	SMOC1	DCMS	Y
	Smurf1	STR	Y
	SOX5	STR	Y
	SYT7	STR	Y
	TBCD	STR	Y
	VEGFA	STR	Y
	XYLT1	DCMS	N
	ZBTB32	STR	Y
**Fat/adipose tissue**	HTR3A	STR	Y
	LRP3	DCMS	Y
	VEGFA	STR	Y
**Vision and olfactory development**	CHD7	SV	Y
	EPHA4	Fixed site	N
	EYE	Fixed site	N
	HS6ST1	Fixed site	N
	MID1	SV, STR, DCMS	Y
	PCDH15	STR	Y
	ROBO1	STR	Y
	ROBO2	DCMS	Y
	SEMA3A	STR	Y
	SOX5	STR	Y
	TOX	Fixed site	N
	VEGFA	STR	Y

Genes belonging to one of the three main categories were found within 8 kb of outlier loci detected by three different types of DNA variation. The DCMS combines five different SNP-based genome statistics commonly used for selection scans, which increases detection power. The last column indicates whether genes were found in significantly enriched pathways found by separately conducted gene ontology analyses. SV, structural variation; STR, short tandem repeat; Y, yes; N, no.

## Discussion

Characterization of the genomic landscape of Arctic populations or subspecies provides important understanding of the potentially adaptive diversity that is associated with this unique and threatened biome. Here, we identified genome-wide outlier loci of SNPs, SVs, and STRs that are divergent between two subspecies of the Atlantic puffin, *F. a. naumann*i and *F. a. arctica*. These loci are in close (ie within 8,000 bp) proximity to genes associated with observed phenotypic differences between the two subspecies, such as body size, skeletal development, or adipose tissue accumulation, as well as genes linked to so far unknown possible physiological differences, such as in the visual and olfactory system. More than 90% of outlier genes were detected by a single type of DNA variation only (SNP, SV, or STR), highlighting the need for including all three types of variants for the identification of potentially adaptive genetic diversity. Our observations yield candidate regions for future research and exemplify how genomic tools can be used to understand genetic differences across populations of nonmodel species.

### Power and Challenges of Combining SNPs, SVs, and STRs

While SNP-based parameters have traditionally been employed to assess genomic differentiation due to their presumed precision and accuracy (Fischer et al. 2017; Prunier et al. 2023), this study highlights their limitations, particularly in cases of recent divergence or weak genetic differentiation, as is common among pelagic seabirds (Lombal et al. 2020). Integrating SV and STR analyses alongside SNP-based assessments provided a more sensitive and comprehensive approach, revealing candidate regions for adaptive divergence that SNP-based methods alone failed to detect. A key finding of this study is that most outlier loci were identified by only one type of genetic marker, emphasizing the complementary nature of these approaches, as previously observed in species such as the American lobster, European starling, and humans (Dorant et al. 2020; Jakubosky et al. 2020; Stuart et al. 2023). STRs, in particular, demonstrated a more significant role in population divergence, including phenotypic differentiation, than traditionally recognized (Fotsing et al. 2019; Reinar et al. 2021, King 2025). Notably, STRs accounted for the highest explained genetic variation between subspecies, contained the greatest proportion of outliers (especially near genes), and were enriched close to genes—suggesting a potential role in gene regulation akin to other study systems (Reinar et al. 2021, 2024; King 2025). Their proximity to a high number of outlier genes and their strong association with genes linked to polar adaptation further suggest that STRs may be critical in shaping adaptive divergence in the Arctic.

While this study represents one of the first to jointly analyze SVs, STRs, and SNPs in a nonmodel species, methodological limitations and potential inherent biases remain. For example, detecting different SV types with short-read sequencing data are affected by methodological biases at varying degrees, influencing their detection rates and necessitating careful interpretation of results (Sedlazeck et al. 2018; Chaisson et al. 2019; Chakraborty et al. 2019; Mahmoud et al. 2019; Zhou et al. 2019; Bertolotti et al. 2020; Pokrovac and Pezer 2022). Additionally, STR mutation rates are orders of magnitude higher than SNP mutation rates (Fan and Chu 2007), and while this could contribute to their greater explanatory power for genetic variation and raise the possibility that a portion of the variation captured by STRs is neutral rather than adaptive (Wellenreuther et al. 2019), recent evidence has demonstrated that STRs are generally not neutral, but under selection and contributing to gene regulation (Reinar et al. 2021, 2024; King 2025). Overall, despite not being infallible, these approaches have been successfully applied across multiple systems, underscoring their utility in comparative and population genomics (Wellenreuther et al. 2019). Hence, our findings advocate for a more holistic genomic framework that integrates multiple types of genetic variation to better understand the mechanisms driving local adaptation and species differentiation (Wellenreuther et al. 2019; Mérot et al. 2020). Future studies should refine these methodologies to mitigate biases and further elucidate the evolutionary significance of different genomic markers.

### SNPs as Indicator for Ancestral Genetic Variation

Combining multiple SNP-based genomic differentiation parameters, such as *F*_ST_, Tajima's *D*, π, or xpEHH, into a single parameter has been shown to be superior to using outliers of genetic divergence or linkage alone (Ma et al. 2015). Specifically evolutionary processes, such as heterogeneity in recombination rates or population stratification, can inflate divergence at loci and introduce bias in *F*_ST_ and other SNP-based outlier methods (Narum and Hess 2011; Bierne et al. 2013; Hoban et al. 2016). Nevertheless, we assessed the relationships between these individual SNP-based parameters, as they have previously been shown to be informative for detecting selection at the whole-genome level (Nachman and Payseur 2012; Cruickshank and Hahn 2014; Ravinet et al. 2017). Here, genome-wide, window-based estimates of absolute divergence (*d_XY_* = 0.0035) closely aligned with the median nucleotide diversity of *naumanni* (π = 0.0033) and *arctica* (π = 0.0035), a pattern also observed in flycatchers (Ellegren et al. 2012; Cruickshank and Hahn 2014). The two Atlantic puffin subspecies are thus genetically not much more different from each other than are individuals within the subspecies, which suggests that most genetic variation in these populations originates from their common ancestor rather than from new mutations or strong genetic drift following divergence. This conclusion is further supported by the strong positive correlation between *d_XY_* and π, with variation in *d_XY_* likely reflecting ancestral polymorphisms rather than extensive postdivergence changes (Noor and Bennett 2009; Cruickshank and Hahn 2014; Henderson and Brelsford 2020). Nevertheless, the relatively recent estimated divergence between *naumanni* and *arctica* (∼15,000 years/1,000 generations ago; Kersten et al. 2023), as well as ongoing gene flow particularly highlighted by the hybrid population in Røst (Kersten et al. 2023), violate the assumptions of migration-drift equilibrium and may hamper the use of absolute divergence estimates in reconstructing recent evolutionary history (Nachman and Payseur 2012; Cruickshank and Hahn 2014; Ravinet et al. 2017). Patterns observed in our data—such as the lack of expected signals from selection at linked sites, selection with gene flow, or ancestral selection—therefore deviate from theoretical expectations under equilibrium conditions (Nachman and Payseur 2012; Cruickshank and Hahn 2014; Guerrero and Hahn 2017; Ravinet et al. 2017). However, the use of composite statistics, such as the DCMS, has proven more robust in detecting selection-driven divergence. In our dataset, DCMS outlier windows exhibited significantly elevated *F*_ST_, alongside notably reduced *d_XY_* and π, particularly in *naumanni*. This pattern suggests potential adaptive divergence between the subspecies (Nachman and Payseur 2012; Ravinet et al. 2017). Interestingly, *d_XY_* was positively correlated with both *F*_ST_ and π within outlier windows, which diverges from expectations under equilibrium models, reinforcing the conclusion that migration-drift equilibrium does not hold in populations of the Atlantic puffin (Nachman and Payseur 2012; Cruickshank and Hahn 2014; Guerrero and Hahn 2017; Ravinet et al. 2017). Collectively, these findings underscore the limitations of relying on individual genomic differentiation parameters in recently diverged populations and confirm the value of composite statistics.

### Outlier Loci are Associated With Bill Shape/Size, Body Size/Fat Tissue, and the Olfactory and Visual System

Combining the DCMS with SV- and STR-based methods we identify multiple loci throughout the puffin genome exhibiting significant divergence between the two subspecies. Several of these outlier loci detected were in close proximity to genes that may be associated with differences in (i) bill shape and size, (ii) body size and fat tissue, and (iii) olfactory and visual system. While differences in the skeletal system, body size, and fat tissue represent traits that are consistent with the known phenotypic differences of the two subspecies (Bosse et al. 2017; Silva et al. 2017; Duntsch et al. 2020), divergence in the visual or olfactory system between the Atlantic puffin subspecies is currently unknown. Additionally, bill shape, body size, and fat tissue accumulation in birds have all previously been linked to adaptation to the polar environment (Blix 2016; McQueen et al. 2022). It is important to note that the observed genetic differentiation is only an association to functional divergence (Vincent et al. 2013). As a first step toward confirmation, differences in nonsynonymous mutations or variation in expression patterns of outlier genes should be investigated. Nevertheless, the close proximity of outlier loci to genes with known functions related to adaptation and phenotypic divergence suggests a potential for adaptive evolution that warrants further investigation, and we discuss their potential functional roles in the following sections.

The first functional group of interest consists of genes that may be associated with the different size and shape of *naumanni* and *arctica* bills (Harris and Wanless 2011; Burnham et al. 2020). This group includes genes like *EP300* (*E1A Binding Protein P300*; Bartsch et al. 2010) and *RAF1* (*Raf-1 Proto-Oncogene, Serine/Threonine Kinase*; Razzaque et al. 2007), which are linked to craniofacial disorders in humans, whereas *PPM1F* (*Protein Phosphatase, Mg2+/Mn2+ Dependent 1F*), *PTPRK* and *NRP2* (*Neuropilin 2*) play potential roles in the bill morphology in birds (Lawson and Petren 2017; Gamboa et al. 2022).

The second functional group of interest consists of genes that might play a role in the larger body size of *naumanni* compared to *arctica* (Salomonsen 1944; Pethon 1967; Harris and Wanless 2011; Burnham et al. 2020) and could be a response to the colder High Arctic habitat of this subspecies (Bergmann 1847; Harris and Wanless 2011; Blix 2016). Here, we detect genes (eg *HTR3A*—*5-Hydroxytryptamine Receptor 3A*, *LRP3*—*LDL Receptor Related Protein 3*, *SMOC1*—*SPARC Related Modular Calcium Binding 1*, *CSMD3*—*CUB And Sushi Multiple Domains 3, GTF3A*—*General Transcription Factor IIIA*) that have been linked with differences in body size, skeletal development, or adipose tissue accumulation in humans, cattle, mice, and frogs (Thomas et al. 2009; Speliotes et al. 2010; Oh et al. 2015; An et al. 2019; Cao et al. 2022). Puffin subspecies divergence in this functional class is supported by the enrichment of the positive regulation of chondrocyte differentiation and growth hormone secretion pathway revealed by the GO analysis.

The third functional group includes genes affecting the olfactory and visual system, with both, divergent loci and fixed SNPs, being in close proximity to genes involved in the development of these systems (Table 1). For example, *ROBO1*, *ROBO2* (*Roundabout Guidance Receptor 1+2*; knockouts of either leads to an improper development of olfactory projections in mice; Fouquet et al. 2007), *CHD7* (*Chromodomain Helicase DNA Binding Protein 7*), and *VEGFA* (*Vascular Endothelial Growth Factor A;* knockout causes vision loss in mice; Kurihara et al. 2012) were identified as outlier genes and are part of the enriched olfactory bulb development and retinal ganglion cell axon guidance pathways. Similarly, fixed SNPs were found in the *EPHA4*, *EYS*, *HS6ST1*, *TOX* genes, which have been associated with retinal nerve development (chickens; Fiore et al. 2019), retinal disease (humans; Yang et al. 2020), olfactory disease (humans; Tornberg et al. 2011), and near-sightedness (humans; Wang et al. 2022), respectively. Yet, these variants are located exclusively within intronic regions, and there is considerable uncertainty regarding whether they can exert any meaningful phenotypic influence. This uncertainty invites the interpretation that these fixed SNPs are better understood as markers of shared evolutionary history rather than as evidence of adaptive significance. Nevertheless, changes and diversity in the olfactory system of birds have previously been assigned to differences in habitat, migratory behavior, foraging strategies, nesting, and sexual selection (Corfield et al. 2015; Khan et al. 2015), and several adaptations of the visual system in birds are linked with motion detection, bright-light, or nonspectral vision (Wu et al. 2016; Martin 2017; Borges et al. 2019; Stoddard et al. 2020). While puffins have photoluminescence bills that potentially play a role in sexual selection requiring a corresponding visual system for detection (Dunning et al. 2018), divergence in the visual or olfactory system between the Atlantic puffin subspecies has to date not been identified (Barrett et al. 2002; Harris and Wanless 2011; Underwood 2019; Anker-Nilssen and Bangjord 2021; Burnham et al. 2021). Hence, our findings highlight new avenues for future research on divergence patterns in nonmodel Arctic species and also provide a foundation for further puffin studies to investigate how the observed phenotypic differences between subspecies may represent adaptations to the High Arctic environment.

### A Note on the Conservation of Puffins in the High Arctic

From a conservation perspective, our findings provide evidence that *F. a. naumanni* on Spitsbergen harbors unique genetic diversity within puffins, and that at least part of this diversity potentially reflects adaptive divergence from the other puffin subspecies. The isolation from the other subspecies in combination with a substantially different habitat that has led to adaptive differences warrants its consideration as a significant evolutionary component of the species as a whole (Funk et al. 2012; Hohenlohe et al. 2021). Given that *naumanni* inhabits the outer edges of the puffin's distribution and that any putative local adaptations will not be easily replenished, the conservation of this subspecies must be a first priority (Hohenlohe et al. 2021). Lastly, the recent detection of a hybrid population between *naumanni* and *arctica* on Bjørnøya (Kersten et al. 2021, 2023) opens up the possibility for investigating the impacts of adaptive introgression (Hamilton and Miller 2016; Campagna and Toews 2022). Since the evolutionary consequences of hybridization range widely, from decreased hybrid fitness to increased levels of genomic diversity promoting the resilience of a species (Todesco et al. 2016; Ottenburghs 2021; Brauer et al. 2023), understanding the patterns of adaptive introgression is particularly relevant. Specifically, research on how subspecies-divergent loci of SNPs, STRs and SVs, as well as their associated genes, are distributed in the hybrids could open up possibilities to gain insights into the fitness effects of a changing climate on a population level and species level (Hamilton and Miller 2016; Campagna and Toews 2022).

## Materials and Methods

### Sampling, Sequencing, and SNP Calling

Samples from a total of 18 (six per colony) adult Atlantic puffin individuals collected across three breeding colonies (Spitsbergen, Bjørnøya, Røst) between 2012 and 2018 were previously sequenced (short read sequencing) to a genome-wide average depth of coverage of 21.6 × (Kersten et al. 2023; Supplementary Data File 1). Spitsbergen was selected as representative colony of the Atlantic puffin subspecies *naumanni*, Røst as representative colony of the subspecies *arctica*, while Bjørnøya is a hybrid population between the two subspecies (Kersten et al. 2021, Figure 1). Samples from Bjørnøya were included to improve the accuracy of calling SNPs, SVs, and STRs by increasing sample size. The detection of outliers was restricted to the samples from Røst and Spitsbergen.

Sequencing reads were processed in PALEOMIX v1.2.14 (Schubert et al. 2014) to generate bam files, as described in Kersten et al. (2023). Genotypes at autosomal single nucleotide polymorphisms (SNPs) were jointly called with GATK v4.2.0 (McKenna et al. 2010) and filtered as in Kersten et al. (2023). The exclusion of allosomes from the analysis was a deliberate choice made to maintain focus on the autosomal genetic architecture and to avoid the complexities associated with sex-linked inheritance patterns as well as haploidy and sample size. Relatedness of individuals was investigated with VCFtools v.0.1.16 (Danecek et al. 2011), which led to the removal of one (of two) related individuals (from Spitsbergen) from the raw, unfiltered and unpruned SNP dataset after evaluation of the KING parameter (Supplementary File 1). Additionally, a SNP panel containing nonvariant sites was produced. This resulted in two datasets: “NoRelatedInd” including 9,907,905 sites, and “NonVarian/NoRelatedInd” comprising 1,000,327,483 sites (Table S3). The SNP genotypes of the NoRelatedInd dataset were used with smartPCA (Patterson et al. 2006) to conduct a principal component analysis (PCA) to validate the genomic population structure identified previously (Kersten et al. 2021, 2023). A more detailed description of laboratory methods and SNP calling can be found in Supplementary File 1.

### SNP-Based Genome Selection Scans

In order to identify genomic regions that significantly differ between the two subspecies, several genomic parameters were estimated in sliding windows along the genomes of Spitsbergen versus Røst individuals and subsequently combined into a single parameter. *F*_ST_, Tajima's *D*, π, and *d_XY_* were calculated in 50 kb sliding windows (25 kb slide) using the “NonVariant/NoRelatedInd” dataset (Table S3). Tajima's *D* was estimated with the utility program VCF-kit (https://vcf-kit.readthedocs.io/en/latest/), while *F*_ST_, π, and *d_XY_* were calculated with the script popgenWindows.py (https://github.com/simonhmartin/genomics_general) for each subspecies and the between-subspecies comparison. To gain insights into genome-wide patterns of selection and local adaptation, correlations between *F*_ST_, *d_XY_*, and π, were calculated using window-based estimates with the Pearson correlation coefficient, while correlations of *F*_ST_, *d_XY_*, and π between outlier and nonoutlier windows were calculated with the Wilcoxon Rank Sum test.

Long-range haplotype estimates were generated after phasing the genomes of all individuals. Specifically, phase sets were identified in each individual genome by WhatsHap v0.18 and used as input for Shapeit4 v4.1 (Delaneau et al. 2019) to statistically phase all genomes. The integrated haplotype homozygosity score (iHS) and the cross-population extended haplotype homozygosity (xpEHH) were calculated on the phased variants for each subspecies and the subspecies comparison, respectively, using the R package rehh (Gautier et al. 2017). Absolute values of iHS and xpEHH were averaged along the genome in sliding windows of 50 kb (slide 25 kb).

Combining estimates of genomic diversity, divergence, and selection into one single parameter, the genome-wide decorrelated composite of multiple signals (DCMS; Ma et al. 2015) was applied. The DCMS combines different statistics for detecting selection signatures while accounting for the correlation between them (Ma et al. 2015). For each 50 kb window, the five aforementioned statistics (iHS, xpEHH, Tajima's *D*, π, and *F*_ST_) were combined to calculate the DCMS with the R package MINOTAUR (Verity et al. 2017), as previously done in multiple selection scan studies (Yurchenko et al. 2018; Ghoreishifar et al. 2020; Zhang et al. 2022). Within-subspecies statistics were obtained from the Spitsbergen genomes, while between-subspecies statistics originated from the Spitsbergen–Røst comparison. *P*-values for each statistic were calculated per window and transformed into a correlation matrix to calculate DCMS values for each window. Significance of DCMS values was tested using a robust linear model, and *P*-values were transformed to *q*-values to control for multiple testing false discovery rate (FDR). Windows with a *q*-value lower than 0.05 were considered statistically significant, and significant overlapping windows were combined to longer (75 kb+) intervals. To evaluate the performance of using only *F*_ST_ windows, we examined the genetic divergence in introns, coding regions, and intergenic regions, and investigated the differences between outlier windows and nonoutlier windows in this regard. Methodological details on the SNP-based genome selection scans are presented in Supplementary File 1.

### SV Outlier Detection

After slightly modifying and extending a previously published SV detection and calling pipeline (Mérot et al. 2023), structural variants (SVs—insertions, deletions, duplications, and inversions) were identified and genotyped using a range of publicly available programs. Delly v0.9.1 (Rausch et al. 2012), Smoove v0.2.8 (https://github.com/brentp/smoove), which is based on Lumpy (Layer et al. 2014), Manta v1.6 (Chen et al. 2016), and Gridss v2.12.2 (Cameron et al. 2021) identified SVs, and Jasmine (Kirsche et al. 2023) merged the results of all four programs. The vg toolkit (Hickey et al. 2020) and Paragraph (Chen et al. 2019) were used to obtain genotype calls for the merged SVs. The four detection programs (Delly, Smoove, Manta, and Gridss) were run with default parameters on all samples jointly, except for Delly, which was run per individual. The output was subsequently filtered as in Mérot et al. (2023) and formatted to make it compatible for the program Jasmine, which merged the output of all four programs. Autosomal SVs that were detected by at least two programs were kept for downstream analyses.

Genotyping of autosomal SVs was performed in two ways. Paragraph called SV genotypes per sample using the recently published Atlantic puffin reference genome (Kersten et al. 2023, GCA_947846985) and information on average read length and depth of coverage of each sample across the genome (Chen et al. 2019). For the other approach, the vg toolkit was used to combine the reference genome with the SV catalog obtained from Jasmine to build a variant-aware genome-graph, followed by mapping sequencing reads and calling SV genotypes. After each approach, biallelic SVs were filtered and genotype calls from both programs were concatenated into a final dataset. Finally, individual SPI002 was removed, as it was not used for any SNP-based analyses due to relatedness with SPI015.

The SV genotypes were used with smartPCA (Patterson et al. 2006) to conduct a PCA assessing the SV-based genomic population structure. *F*_ST_ values for each SV between Spitsbergen and Røst individuals were calculated with VCFtools v0.1.16 and converted to *P*-values with the R package MASS (Ripley et al. 2013). *P*-values were subsequently transformed to *q*-values to control for multiple testing FDR. SVs with a *q*-value lower than 0.05 were considered as statistically significant outliers. Finally, to ensure the quality of the SV data, the relationships between sequencing depth and SV length, as well as between *F*_ST_ and missingness, were investigated. More information on SV detection and genotyping can be found in Supplementary File 1.

### STR Outlier Detection

STRs were identified and genotyped along the genomes of the 18 puffins with HipSTR v0.6.2 (Willems et al. 2017) following Reinar et al. (2021). A HipSTR specific genome-wide catalogue of STRs was built with TandemRepeatsFinder v4.09 (Benson 1999) followed by filtering and calling genotypes with HipSTR. A final autosomal STR genotype panel was created by only retaining STRs that were called in all individuals and had a minimum minor allele frequency of >0.1, and by removing individual SPI002. This individual was removed, as it was not used for any SNP-based analyses due to relatedness with SPI015.

STR genotypes were used in a PCA performed by smartPCA (Patterson et al. 2006) to validate the genomic population structure identified with SNPs (Kersten et al. 2023). Outlier STRs between the *naumanni* and *arctica* subspecies were discovered with significantly high genomic differentiation as assessed by values of Jost's *D* (Jost 2008). Jost's *D* was calculated with the R package *mmod* (https://github.com/dwinter/mmod) and values were converted to *P*-values with the R package MASS. *P*-values were subsequently transformed to *q*-values to control for FDR. STRs with a *q*-value lower than 0.05 were considered as statistically significant outliers. More details on the STR outlier detection methods are listed in Supplementary File 1.

### Outlier Genes and Fixed Single Nucleotide Polymorphisms

Protein-coding genes that fell within (1 bp overlap) 8 kb of DCMS (window-based), STR and SV (both point-based) outliers were designated as “outlier genes.” After exploring the distribution of distances from outlier and nonoutlier DCMS windows to the nearest CDS, a distance of 8 kb was chosen as it represents half the average distance between two protein-coding genes in the new puffin reference genome annotation. A targeted literature review was conducted to assemble a list of candidate genes potentially involved in traits relevant to the environmental or phenotypic differences across *arctica* and *naumanni*. Publications were manually screened for studies that identified genes in birds or other polar-adapted fauna associated with morphological traits (eg beak size and shape), cold adaptation, skeletal development (eg ossification and cranial morphology), and tissue-specific processes (eg adipose tissue development and thermogenesis). Studies using genome-wide approaches such as outlier detection and or genome-wide association studies were prioritized, as these methods often provide statistical support for links between genotype and adaptive phenotype. Keywords including “cold adaptation,” “morphology,” “beak size,” “skeletal development,” “GWAS,” “outlier analysis,” and specific taxa names (eg “penguin” and “polar bear”) were used to search databases such as PubMed and Google Scholar. From these studies (Abzhanov et al. 2004; Zhang et al. 2014; Huntley et al. 2015; Lamichhaney et al. 2015, 2016; Lowe et al. 2015; Mason and Taylor 2015; Ravinet et al. 2018; Li et al. 2019; Sendell-Price et al. 2020; Walsh 2021; Pirri et al. 2022; Gamboa et al. 2022), genes highlighted as candidates due to significant associations or repeated identification across taxa were compiled into the reference list (Supplementary Data File 1). Moreover, genes found via a QuickGo (https://www.ebi.ac.uk/QuickGO/) search using the keywords “adipose tissue development,” “muscle adaptation,” “response to cold,” “adaptive thermogenesis,” “temperature homeostasis,” “cranial skeletal system development,” and “skeletal system development” were added to the reference list. Outlier genes were then cross-referenced against this manually compiled and curated list of 2,275 unique genes (Supplementary Data File 1). Outlier genes were also run through two GO analyses with ClueGO v2.5.9 (Bindea et al. 2009), using a human (*Homo sapiens*) and a chicken (*Gallus gallus*) gene set.

Sites for which the alternative SNP allele was fixed in all Spitsbergen individuals were further investigated. The positions of these SNPs were cross-referenced against the puffin genome annotation and manually inspected, and their effect was assessed with snpEff v4.3 (Cingolani et al. 2012). A thorough description of the outlier gene and fixed SNP analyses are provided in Supplementary File 1.

## Supplementary Material

evaf162_Supplementary_Data

## Data Availability

Raw read data analyzed in the current study have been deposited in the European Nucleotide Archive (ENA, www.ebi.ac.uk/ena) under study accession number PRJEB40631 (see Supplementary Data File 1 for individual sample accession numbers and metadata). All code used for the genomic analyses are available on the first author's GitHub (https://github.com/OKersten/DCMS_SV_STR_Outliers) and on Zenodo under https://doi.org/10.5281/zenodo.7531163. This includes versions of any software used, if relevant, and any specific variables or parameters used to generate, test, and process the dataset of this study.
