# Supplementary material for: Single Nucleotide Polymorphisms, Structural Variants, and Short Tandem Repeats Capture Distinct Signals of Adaptive Divergence in the Atlantic Puffin
Source: Genome Biol Evol. 2025 Aug 19;17(9):evaf162. doi: 10.1093/gbe/evaf162 (PMC12455043; doi:10.1093/gbe/evaf162)
Supplement: evaf162_Supplementary_Data [file evaf162_supplementary_data.zip › SupplementaryFile1_ReReResub.pdf]

Single nucleotide polymorphisms, structural variants and short tandem repeats capture distinct signals of adaptive divergence in the Atlantic puffins

Oliver Kersten[1]*, Bastiaan Star[1], Tycho Anker-Nilssen[2], Hallvard Strøm[3], Kjetill S. Jakobsen[1], Sanne Boessenkool[1]*

[1] *Centre for Ecological and Evolutionary Synthesis (CEES), Department of Biosciences, University of Oslo, Oslo, Norway*
[2]*Norwegian Institute for Nature Research (NINA), Trondheim, Norway*
[3]*Norwegian Polar Institute, Fram Centre, Langnes, Tromsø, Norway*

corresponding authors: okerstenwork@gmail.com, sanne.boessenkool@ibv.uio.no

1. ***SUPPLEMENTARY METHODS***
2. ***SUPPLEMENTARY TABLES***
3. ***SUPPLEMENTARY FIGURES***

1. SUPPLEMENTARY METHODS

1.1 Sampling, Sequencing and Data Processing

Samples from a total of 18 puffins collected across three breeding colonies were made available for the present study by SEAPOP (http://www.seapop.no/en) and SEATRACK (http://www.seapop.no/en/seatrack/). These samples had been collected between 2012-2018 and consisted of blood preserved in EtOH or lysis buffer, or feathers. They were sequenced for a previous whole-genome Atlantic puffin study (Kersten et al., 2023) to a genome-wide average depth of coverage of 21.6 (Supplementary Data File 1). Spitsbergen was selected as representative colony of the Atlantic puffin subspecies *naumanni*, Røst as representative colony of the subspecies *arctica*, and Bjørnøya was previously identified as hybrid zone between the two subspecies (Kersten et al. 2021). DNA from these samples was extracted as described in Kersten et al. (2021). Samples from Bjørnøya were included for calling SNPs, SVs and STRs in order to increase samples size and with that power of the variant calling, but comparative analyses between the two subspecies were performed between Røst and Spitsbergen only.

The 18 genomic libraries, which were initially built for and part of the analysis of Kersten et al. (2021), were resequenced across four lanes on an Illumina HiSeq4000 (as detailed in Kersten et al., 2023). Sequencing reads were processed in PALEOMIX v1.2.14 (Schubert et al. 2014), mapped to the new Atlantic puffin assembly using BWA *mem* v0.7.17 (Li 2013) and split into nuclear and mitochondrial bam files using SAMtools v1.9 (Li et al. 2009) as described in Kersten et al. (2023).

1.2 SNP-based analyses

1.2.1 SNP calling and filtering

Genotypes at autosomal single nucleotide polymorphisms (SNPs) were jointly called with GATK v4.2.0 (McKenna et al. 2010) by using the *HaplotypeCaller*, *GenomicsDBImport* and *GenotypeGVCFs* tools. Genotypes were filtered with BCFtools v1.9 (Li et al. 2009) by applying *“--SnpGap 10 -e 'QD < 2.0 || MQ < 40 || FS > 60.0 || SOR > 3 || 12.5 > MQRankSum < -12.5 || 8> ReadPosRankSum < -8.0'”* according to GATKs Best Practices (Van der Auwera et al. 2013) and genotypes with a read depth less than 3 or a quality less than 15 were set as missing. Using BCFtools v1.9 and VCFtools v.0.1.16 (Danecek et al. 2011), indels and non-biallelic SNPs were removed and only sites with a minimum quality of 30, global depth of 250-500X and a mean depth of 10.8-43.2X (0.5 and 2x of overall mean depth) across the 18 samples were kept. Finally, only SNPs present in all individuals were retained for subsequent analyses.

1.2.2 Relatedness

Using the resulting SNP dataset, relatedness between individuals was investigated with VCFtools v.0.1.16 (*--relatedness2*), which uses the KING inference (Manichaikul et al. 2010) to find probabilities of relatedness. An estimated kinship coefficient range >0.354, 0.177-0.354, 0.0884-0.177 and 0.0442-0.0884 corresponds to duplicate/MZ twin, 1st-degree, 2nd-degree, and 3rd-degree relationships, respectively (Manichaikul et al. 2010). After detecting two related (2nd-degree) individuals on Spitsbergen (SPI002 and SPI015), SPI002 was removed from the raw, unfiltered and unpruned SNP dataset as it had the lower overall depth of coverage across the genome (20.04X vs. 21.59X, Supplementary Data File 1). Subsequently, all SNP filtering was

repeated as detailed above (note: some filter values changed, e.g. mean depth of 10.9-43.3X), resulting in a SNP dataset containing 17 individuals and 9,907,905 sites.

1.2.3 Non-variant sites and outgroup

In addition to the SNP dataset generated above, a SNP panel containing non-variant sites was produced. Applying the flag "*--include-non-variant-sites*" during GATK *GenotypeGVCFs* and the same filters outlined above, the "NonVariant//NoRelatedInd" (17 ind., 1,000,327,483 sites) dataset was generated.

1.2.4 Genome selection scans

In order to identify genomic regions that significantly differ between the two subspecies, we estimated a wide range of parameters in sliding windows along the genome between Spitsbergen and Røst individuals and subsequently combined them into a single parameter.

First, F_{ST}, Tajima's D, nucleotide diversity (**π**) and d_{XY} were calculated in 50 kb sliding windows (25 kb slide) using the "NonVariant/NoRelatedInd" dataset. Tajima's D was estimated for each population with the *tajima* function (vk tajima 50,000 25,000) of the utility program VCF-kit (https://vcf-kit.readthedocs.io/en/latest/). F_{ST}, **π** and d_{XY} were calculated with the script *popgenWindows.py* (-w 50000 -m 10 -s 25000; written by Simon Martin - https://github.com/simonhmartin/genomics_general) for each population and the between-population comparison.

Subsequently, long-range haplotype estimates were generated after phasing the genomes of all individuals. Specifically, using the "NoRelatedInd" dataset, phase sets were identified in each individual puffin genome by WhatsHap v0.18 (--tag=PS; Patterson et al. 2015). The phase sets were then used as input for Shapeit4 v4.1 (Delaneau et al. 2018) to statistically phase (--use-PS 0.0001) all puffin genomes. The integrated haplotype homozygosity score (iHS) and the cross-population extended haplotype homozygosity (xpEHH) were calculated on the phased variants for each subspecies and the subspecies comparison, respectively, using R v4.1 (R Core Team 2020) and the package *rehh* (Gautier et al. 2016). Absolute values of iHS and xpEHH were then averaged along the genome in sliding windows of 50 kb (slide 25 kb).

Combining estimates of genomic diversity, divergence and selection into one single parameter, the genome-wide de-correlated composite of multiple signals (DCMS; Ma et al. 2015) was applied. The DCMS combines different statistics for detecting selection signatures while accounting for the correlation between the different selection signature statistics and has shown to have a higher power than most of the single statistics (Ma et al. 2015). For each 50 kb window, the five above-mentioned statistics (iHS, xpEHH, Tajima's D, **π**, and F_{ST}) were combined to calculate the DCMS in R v3.6 (R Core Team 2020) with the *MINOTAUR* package (Verity et al. 2017), as previously done in multiple selection scan studies (Yurchenko et al. 2018, Ghoreishifar et al. 2020, Zhang et al. 2022). Within-population statistics were taken from the Spitsbergen genomes, while between-population statistics originated from the Spitsbergen-Røst comparison. To calculate the DCMS, p-values for each statistic were calculated for each window by applying the *stat_to_pvalue* function of *MINOTA*UR including unilateral left tailed tests for the **π** and Tajima's D statistics and right tailed tests for the |iHS|, |xpEHH| and F_{ST} statistics. Subsequently an n x n correlation matrix between the included statistics was constructed by calling the *covNAMcd* function (alpha = 0.75, nsamp = 50,000) from the *rrcovNA* R package (Todorov et al.

2011). After calculating DCMS values for each window with the *DCMS* function of the *MINOTAUR* package using the correlation matrix as input, they were fitted to a normal distribution with a robust linear model using the rlm function (dcms ~ 1) of the R package *MASS* (Ripley et al. 2013). The mean and standard deviation of the fitting model were supplied to the *pnorm* R function to calculate the p-value of the DCMS value of each window. P-values were then transformed to q-values to control for multiple testing false discovery rate (FDR) by applying the *p.adjust* R function and using the Benjamini-Hochberg correction. Windows with a q-value lower than 0.05 were considered as statistically significant and significant overlapping windows were combined to longer (75kb+) intervals. Significance of correlations of F_{ST}, π and d_{XY} was established with Pearson correlation tests (Pearson 1895) and significance of difference in the three parameters between outlier and non-outlier windows was assessed with Mann-Whitney U tests (Mann and Whitney 1947).

1.3 Genomic architecture analyses

1.3.1 Structural Variant (SV) detection and genotyping

After slightly modifying and extending a recently published SV detection and calling pipeline (Merot et al. 2022), SVs (insertions, deletions, duplications, and inversions) were identified and genotyped using a diverse suite of publicly available programs. Delly v0.9.1 (Rausch et al. 2012), Smoove v0.2.8 (https://github.com/brentp/smoove), which is based on Lumpy (Layer et al. 2014), Manta v1.6 (Chen et al. 2016) and Gridss v2.12.2 (Cameron et al. 2021) identified SVs, and Jasmine (Kirsche et al. 2021) merged the results of all four programs. The vg toolkit (Hickey et al. 2020) and Paragraph (Chen et al. 2019) were used to obtain genotype calls for the merged SVs. Initially, all sequence data needed to be remapped in Paleomix without the GATK realignment step, as Gridss doesn't support realigned GATK bam files (Cameron et al. 2021). Additionally, realigned (Delly, Smoove, Manta) and non-realigned (Gridss) bam files were soft-clipped with BamUtil/1.0.14 (Breese and Liu 2013).

All four programs were run with default parameters on all samples jointly, except for Delly, which was run per individual. The output of Gridss needed to be altered with the script *simple-event-annotation.R* (https://github.com/PapenfussLab/gridss/blob/master/example/simple-event-annotation.R) to convert unresolved breakpoints (BNDs) to insertions (INS), deletions (DEL), inversions (INV), and duplications (DUP) for all downstream analyses. The results of all four SV identification programs were further filtered by removing translocations (TRAs), BNDs, SVs that overlapped a gap of 10 Ns, and SVs with missing sequence information (Merot et al. 2022), as well as by applying additional filters suggested by the original developers of each program. The format of each output file was then adjusted in a similar way as in Merot et al. (2022) to make it compatible for the program Jasmine. The output of all four programs was joined with Jasmine with the parameters "--ignore_strand --default_zero_genotype --mutual_distance --max_dist_linear=0.5 --max_dist=1000 --min_dist=50 --use_end --normalize_type --allow_intrasample". Autosomal SVs that were detected by at least two programs were kept for downstream analyses.

Genotyping of autosomal SVs was performed in two ways. After formatting the input SV catalog obtained from Jasmine, Paragraph called SV genotypes per sample using the Atlantic

puffin reference genome sequence (Kersten et al. 2023) and information on average read length and depth of coverage of each sample across the genome. The maximum depth cutoff was set to 20x the average depth of coverage per individual (Chen et al. 2019). For the other approach, the reference genome was combined with the SV catalog obtained from Jasmine to build a variant-aware genome-graph with the vg toolkit module *vg autoindex*. Sequencing reads from the 18 individuals were then mapped to the graph with *vg giraffe* (Sirén et al. 2021) and genotypes called with the vg toolkit module *vg call.* After each method, biallelic SVs that were covered by at least one read, genotyped in >50% of individuals and polymorphic in more than one sample were kept for subsequent analyses followed by removing SPI002 (one of the two related individuals) from the dataset. The genotype calls from the vg toolkit and Paragraph were then concatenated with BCFtools v1.9 into a final dataset containing genotypes of SVs across 17 individuals.

1.3.2 Genomic differentiation and outlier detection based on structural variants

The SV genotypes were used in a PCA to validate the genomic population structure identified with SNPs (Kersten et al. 2023). *SmartPCA* (Patterson et al. 2006) of the Eigensoft package was run using "*outliermode: 2, altnormstyle: yes, lsqproject: yes*", followed by investigating the eigenvectors and eigenvalues of the significant principal components as revealed by the Tracy-Widom statistics.

Outlier SVs showing significantly high genomic differentiation between *naumanni* and *arctica* populations were identified. F_{ST} values for each SV between Spitsbergen and Røst individuals were calculated with VCFtools v0.1.16 (*--weir-fst-pop rost --weir-fst-pop spitsbergen*) and fitted to a normal distribution with a robust linear model using the rlm function (F_{ST} ~ 1) of the R package *MASS* (Ripley et al. 2013). The mean and standard deviation of the fitting model were supplied to the *pnorm* R function to calculate the p-value of the F_{ST} values for the SVs along the genome. P-values were subsequently transformed to q-values to control for multiple testing false discovery rate (FDR) by applying the *p.adjust* R function and using the Benjamini-Hochberg correction. SVs with a q-value lower than 0.05 were considered as statistically significant outliers.

1.3.3 Short Tandem Repeat (STR) detection and genotyping

Short Tandem Repeats (STRs) were identified and genotyped along the genomes of the 18 puffins with HipSTR v0.6.2 (Willems et al. 2017) using previously published methodology (Reinar et al. 2021) as guidance. A HipSTR specific genome-wide dataset of STRs was built with TandemRepeatsFinder v4.09 (Benson 1999) following filters and instructions of the HipSTR manual for non-human HipSTR references (https://github.com/HipSTR-Tool/HipSTR-references/). Mapped sequencing data was supplied as input to HipSTR to perform PCR-stutter aware genotype calling of STRs and calls were filtered according to default instructions (https://github.com/HipSTR-Tool/). A final autosomal STR genotype dataset was created by only retaining STRs that were called in all individuals and had a minimum minor allele frequency of >0.1, and by removing individual SPI002.

1.3.4 Genomic differentiation and outlier detection based on short tandem repeats

The STR genotypes were used in a PCA to validate the genomic population structure identified with SNPs (Kersten et al. 2023). Genotypes were converted to a microsatellite input format for *smartPCA* as suggested by Patterson et al. (2006). *SmartPCA* was run using "*outliermode: 2,*

altnormstyle: yes, lsqproject: yes", followed by investigating the eigenvectors and eigenvalues of the significant principal components as revealed by the Tracy-Widom statistics.

Outlier STRs showing significantly high genomic differentiation between the Spitsbergen and Røst populations were discovered using values of Jost's D (Jost 2008), which is suitable for multiallelic data (Jost et al. 2018). Jost's D values were calculated with the R package *mmod* (https://github.com/dwinter/mmod) and fitted to a normal distribution with a robust linear model using the rlm function (JostD ~ 1) of the R package *MASS* (Ripley et al. 2013). The mean and standard deviation of the fitting model were supplied to the *pnorm* R function to calculate the p-value of the Jost's D values for the STRs along the genome. P-values were subsequently transformed to q-values to control for the FDR by applying the *p.adjust* R function and using the Benjamini-Hochberg correction. STRs with a q-value lower than 0.05 were considered as statistically significant outliers.

1.4 Outlier Genes and fixed polymorphisms

Protein coding genes within 8kb (half the average distance between two protein coding genes in the new puffin reference genome annotation) of DCMS outlier windows, STR outliers and SV outliers, hereinafter referred to as "outlier genes", were identified. These genes were cross-referenced against a manually compiled and curated list of 2,275 unique genes that have been shown to be associated with morphology, (e.g. beak size), cold adaptation, skeletal processes (e.g. ossification and cranial development), or tissue adaptation (e.g. adipose tissue development) (Abzhanov et al. 2004, Huntley et al. 2014, Lowe et al. 2014, Zhang et al. 2014, Lamichhaney et al. 2015, 2016, Mason and Taylor 2015, Ravinet et al. 2018, Li et al. 2019, Sendell-Price et al. 2020, Pirri et al. 2021, Walsh 2021, Gamboa et al. 2022).

Outlier genes were also run through two gene ontology (GO) analyses with ClueGO v2.5.9 (Bindea et al. 2009) in Cytoscape 3.9.1 (Shannon et al. 2003). The analyses were based on biological processes pathways only (updated 25.05.2022) and applied parameters included GO Term Fusion, medium network specificity, a right-sided hypergeometric test, Benjamini-Hochberg FDR correction and a q-value of < 0.05. One GO analysis was conducted on a human (*Homo sapiens*) gene set, the other on a chicken (*Gallus gallus*) gene set.

Sites that presumably reached fixation in the Spitsbergen population were further investigated. All SNPs in the "NoRelatedInd" dataset, for which all Røst individuals were homozygous carrying the reference allele and all Spitsbergen individuals were homozygous carrying the alternate allele, were identified. The positions of SNPs were then cross-referenced against the puffin genome annotation and manually inspected. Their effect was assessed with snpEff v4.3 (Cingolani et al. 2012).

2. References

Abzhanov A, Protas M, Grant BR, Grant PR, Tabin CJ (2004). Bmp4 and morphological variation of beaks in Darwin's finches. Science 305: 1462–1465.

Benson G (1999). Tandem repeats finder: a program to analyze DNA sequences. Nucleic Acids Res 27: 573–580.

Bindea G, Mlecnik B, Hackl H, Charoentong P, Tosolini M, Kirilovsky A, et al. (2009). ClueGO: a Cytoscape plug-in to decipher functionally grouped gene ontology and pathway annotation networks. Bioinformatics 25: 1091–1093.

Breese MR, Liu Y (2013). NGSUtils: a software suite for analyzing and manipulating next-generation sequencing datasets. Bioinformatics 29: 494–496.

Cameron DL, Baber J, Shale C, Valle-Inclan JE, Besselink N, van Hoeck A, et al. (2021). GRIDSS2: comprehensive characterisation of somatic structural variation using single breakend variants and structural variant phasing. Genome Biol 22: 202.

Chen S, Krusche P, Dolzhenko E, Sherman RM, Petrovski R, Schlesinger F, et al. (2019). Paragraph: a graph-based structural variant genotyper for short-read sequence data. Genome Biol 20: 291.

Chen X, Schulz-Trieglaff O, Shaw R, Barnes B, Schlesinger F, Källberg M, et al. (2016). Manta: rapid detection of structural variants and indels for germline and cancer sequencing applications. Bioinformatics 32: 1220–1222.

Cingolani P, Platts A, Wang LL, Coon M, Nguyen T, Wang L, et al. (2012). A program for annotating and predicting the effects of single nucleotide polymorphisms, SnpEff: SNPs in the genome of Drosophila melanogaster strain w1118; iso-2; iso-3. Fly 6: 80–92.

Danecek P, Auton A, Abecasis G, Albers CA, Banks E, DePristo MA, et al. (2011). The variant call format and VCFtools. Bioinformatics 27: 2156–2158.

Delaneau O, Zagury J-F, Robinson M, Marchini J, Dermitzakis E (2018). Integrative haplotype estimation with sub-linear complexity. bioRxiv: 493403.

Gamboa MP, Ghalambor CK, Scott Sillett T, Morrison SA, Chris Funk W (2022). Adaptive divergence in bill morphology and other thermoregulatory traits is facilitated by restricted gene flow in song sparrows on the California Channel Islands. Mol Ecol 31: 603–619.

Gautier M, Klassmann A, Vitalis R (2017). rehh 2.0: a reimplementation of the R package rehh to detect positive selection from haplotype structure. Mol Ecol Resour 17: 78–90.

Ghoreishifar SM, Eriksson S, Johansson AM, Khansefid M, Moghaddaszadeh-Ahrabi S, Parna N, et al. (2020). Signatures of selection reveal candidate genes involved in economic traits and cold acclimation in five Swedish cattle breeds. Genet Sel Evol 52: 52.

Hickey G, Heller D, Monlong J, Sibbesen JA, Sirén J, Eizenga J, et al. (2020). Genotyping structural variants in pangenome graphs using the vg toolkit. Genome Biol 21: 35.

Huntley RP, Sawford T, Mutowo-Meullenet P, Shypitsyna A, Bonilla C, Martin MJ, et al. (2015). The GOA database: gene Ontology annotation updates for 2015. Nucleic Acids Res 43: D1057–63.

Jost L (2008). G(ST) and its relatives do not measure differentiation. Mol Ecol 17: 4015–4026.

Jost L, Archer F, Flanagan S, Gaggiotti O, Hoban S, Latch E (2018). Differentiation measures for conservation genetics. Evol Appl 11: 1139–1148.

Kersten O, Star B, Leigh DM, Anker-Nilssen T, Strøm H, Danielsen J, et al. (2021). Complex population structure of the Atlantic puffin revealed by whole genome analyses. Communications Biology 4: 922.

Kersten, O., Star, B., Krabberød, A. K., Atmore, L. M., Tørresen, O. K., Anker-Nilssen, T., Descamps, S., Strøm, H., Ulf S. Johansson, Sweet, P. R., Jakobsen, K. S., & Boessenkool, S. (2023). Hybridization of Atlantic puffins in the Arctic coincides with 20th-century climate change. Science Advances, 9, https://doi.org/10.1126/sciadv.adh1407

Kirsche M, Prabhu G, Sherman R, Ni B, Aganezov S, Schatz MC (2021). Jasmine: Population-scale structural variant comparison and analysis. bioRxiv: 2021.05.27.445886.

Lamichhaney S, Berglund J, Almén MS, Maqbool K, Grabherr M, Martinez-Barrio A, et al. (2015). Evolution of Darwin's finches and their beaks revealed by genome sequencing. Nature 518: 371–375.

Lamichhaney S, Han F, Berglund J, Wang C, Almén MS, Webster MT, et al. (2016). A beak size locus in Darwin's finches facilitated character displacement during a drought. Science 352: 470–474.

Layer RM, Chiang C, Quinlan AR, Hall IM (2014). LUMPY: a probabilistic framework for structural variant discovery. Genome Biol 15: R84.

Li H (2013). Aligning sequence reads, clone sequences and assembly contigs with BWA-MEM. arXiv preprint arXiv:13033997.

Li H, Handsaker B, Wysoker A, Fennell T, Ruan J, Homer N, et al. (2009). The sequence alignment/map format and SAMtools. Bioinformatics 25: 2078–2079.

Li D, Li Y, Li M, Che T, Tian S, Chen B, et al. (2019). Population genomics identifies patterns of genetic diversity and selection in chicken. BMC Genomics 20: 263.

Lowe CB, Clarke JA, Baker AJ, Haussler D, Edwards SV (2014). Feather Development Genes and Associated Regulatory Innovation Predate the Origin of Dinosauria. Mol Biol Evol 32: 23–28.

Ma Y, Ding X, Qanbari S, Weigend S, Zhang Q, Simianer H (2015). Properties of different selection signature statistics and a new strategy for combining them. Heredity 115: 426–436.

Manichaikul A, Mychaleckyj JC, Rich SS, Daly K, Sale M, Chen W-M (2010). Robust relationship inference in genome-wide association studies. Bioinformatics 26: 2867–2873.

Mann HB, Whitney DR (1947). On a test of whether one of two random variables is stochastically larger than the other. Ann Math Stat 18: 50–60.

Mason NA, Taylor SA (2015). Differentially expressed genes match bill morphology and plumage despite largely undifferentiated genomes in a Holarctic songbird. Mol Ecol 24: 3009–3025.

McKenna A, Hanna M, Banks E, Sivachenko A, Cibulskis K, Kernytsky A, et al. (2010). The Genome Analysis Toolkit: a MapReduce framework for analyzing next-generation DNA sequencing data. Genome Res 20: 1297–1303.

Mérot C, Stenløkk KSR, Venney C, Laporte M, Moser M, Normandeau E, et al. (2022). Genome assembly, structural variants, and genetic differentiation between lake whitefish young species pairs (Coregonus sp.) with long and short reads. Mol Ecol.

Patterson M, Marschall T, Pisanti N, van Iersel L, Stougie L, Klau GW, et al. (2015). WhatsHap: Weighted Haplotype Assembly for Future-Generation Sequencing Reads. J Comput Biol 22: 498–509.

Patterson N, Price AL, Reich D (2006). Population structure and eigenanalysis. PLoS Genet 2: e190.

Pearson K (1895). Notes on regression and inheritance in the case of two parents. Proc R Soc Lond 58: 240–242.

Pirri F, Ometto L, Fuselli S, Fernandes FAN, Ancona L, Le Bohec C, et al. (2021). Selection-driven adaptation to the extreme Antarctic environment in the Emperor penguin. bioRxiv: 2021.12.14.471946.

Rausch T, Zichner T, Schlattl A, Stütz AM, Benes V, Korbel JO (2012). DELLY: structural variant discovery by integrated paired-end and split-read analysis. Bioinformatics 28: i333–i339.

Ravinet M, Elgvin TO, Trier C, Aliabadian M, Gavrilov A, Sætre G-P (2018). Signatures of human-commensalism in the house sparrow genome. Proc Biol Sci 285.

R Core Team (2020). R: A language and environment for statistical computing.

Reinar WB, Lalun VO, Reitan T, Jakobsen KS, Butenko MA (2021). Length variation in short tandem repeats affects gene expression in natural populations of Arabidopsis thaliana. Plant Cell 33: 2221–2234.

Ripley B, Venables B, Bates DM, Hornik K, Gebhardt A, Firth D, et al. (2013). Package 'mass'. Cran r 538: 113–120.

Schubert M, Ermini L, Der Sarkissian C, Jónsson H, Ginolhac A, Schaefer R, et al. (2014). Characterization of ancient and modern genomes by SNP detection and phylogenomic and metagenomic analysis using PALEOMIX. Nature Protocols 9: 1056–1082.

Sendell-Price AT, Ruegg KC, Clegg SM (2020). Rapid morphological divergence following a human-mediated introduction: the role of drift and directional selection. Heredity 124: 535–549.

Shannon P, Markiel A, Ozier O, Baliga NS, Wang JT, Ramage D, et al. (2003). Cytoscape: a software environment for integrated models of biomolecular interaction networks. Genome Res 13: 2498–2504.

Sirén J, Monlong J, Chang X, Novak AM, Eizenga JM, Markello C, et al. (2021). Genotyping common, large structural variations in 5,202 genomes using pangenomes, the Giraffe mapper, and the vg toolkit. bioRxiv: 2020.12.04.412486.

Todorov V, Templ M, Filzmoser P (2011). Detection of multivariate outliers in business survey data with incomplete information. Adv Data Anal Classif 5: 37–56.

Van der Auwera GA, Carneiro MO, Hartl C, Poplin R, Del Angel G, Levy-Moonshine A, et al. (2013). From FastQ data to high confidence variant calls: the Genome Analysis Toolkit best practices pipeline. Curr Protoc Bioinformatics 43: 11.10.1–33.

Verity R, Collins C, Card DC, Schaal SM, Wang L, Lotterhos KE (2017). minotaur: A platform for the analysis and visualization of multivariate results from genome scans with R Shiny. Mol Ecol Resour 17: 33–43.

Walsh G (2021). Whole genome data provides evidence of divergent selection and gene flow between two populations of red grouse Lagopus lagopus scotica with implications ….

Willems T, Zielinski D, Yuan J, Gordon A, Gymrek M, Erlich Y (2017). Genome-wide profiling of heritable and de novo STR variations. Nat Methods 14: 590–592.

Yurchenko AA, Daetwyler HD, Yudin N, Schnabel RD, Vander Jagt CJ, Soloshenko V, et al. (2018). Scans for signatures of selection in Russian cattle breed genomes reveal new candidate genes for environmental adaptation and acclimation. Sci Rep 8: 12984.

Zhang G, Li C, Li Q, Li B, Larkin DM, Lee C, et al. (2014). Comparative genomics reveals insights into avian genome evolution and adaptation. Science 346: 1311–1320.

Zhang S, Yao Z, Li X, Zhang Z, Liu X, Yang P, et al. (2022). Assessing genomic diversity and signatures of selection in Pinan cattle using whole-genome sequencing data. BMC Genomics 23: 460.

2. SUPPLEMENTARY TABLES

Table S1: Fixed genetic differentiation between two Atlantic puffin (*Fratercula arctica*) subspecies - *F. a. naumanni* and *F. a. arctica*. Single nucleotide polymorphisms are point based whereas structural variants and short tandem repeats are window based. “Fixed” refers to the scenario where all *F. a. arctica* individuals are homozygous for the reference allele and all *F. a. naumanni* individuals are homozygous for the alternate allele.

Type of Genetic Variation	Total No. of Differences	No. of Fixed Differences			
		Total	Within Genes	Synonymous	Non-Synonymous
**Single Nucleotide Polymorphism**	9,907,905	23	12	0	0
**Structural Variant**	8,640	0	0	0	0
**Short Tandem Repeat**	19,260	0	0	0	0

Table S2: Subset of significantly enriched biological pathways and associated genes found in close proximity to divergent loci detected between two Atlantic puffin subspecies. This subset was selected according to the interest in pathways potentially involved in the phenotypic differences observed between *F. a. arctica* and *F. a. naumanni.* Outlier loci were detected with single nucleotide polymorphisms, structural variants, and short tandem repeats. Genes found within 8 kb of outliers were subsequently used for a gene ontology analysis.

**ID**	**Term**	**Associated Genes Found**
GO:0032332	positive regulation of chondrocyte differentiation	[BMPR1B, RUNX2, SOX5]
GO:0021772	olfactory bulb development	[CHD7, ROBO1, ROBO2, SEMA3A]
GO:0031290	retinal ganglion cell axon guidance	[BMPR1B, ROBO2, VEGFA]
GO:0030252	growth hormone secretion	[ARHGEF7, CHD7, GHSR, KALRN]
GO:0060123	regulation of growth hormone secretion	[ARHGEF7, CHD7, GHSR, KALRN]

Table S3: Single nucleotide polymorphism datasets used for different Atlantic puffin population genomic analyses. All datasets were generated mapping high-coverage (>20X) sequencing data and calling SNPs with GATK v4.2.0.

Name	No. Of Individuals	No. Of Sites	Analyses
NoRelatedInd	17	9,907,905	Phasing, LongRange Haplotype, Fixed Sites
NonVariant/NoRelatedInd	17	1,000,327,483	FST, Tajima's D, nucelotide diversity

3. SUPPLEMENTARY FIGURES

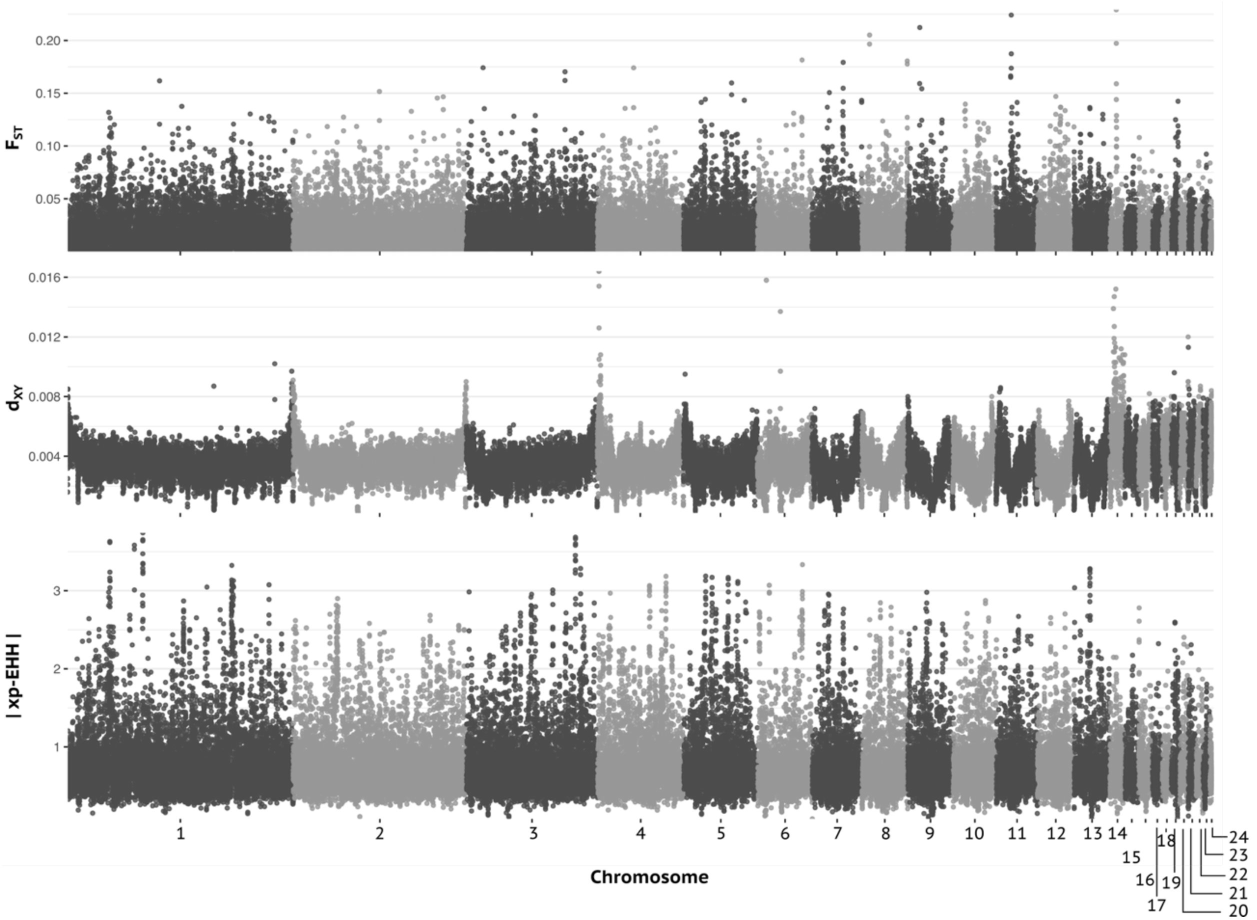

Figure S1: Autosomal distribution of SNP-based measures of between-population divergence between two Atlantic puffin subspecies. Relative (F_{ST}) and absolute (d_{XY}) genetic differentiation, as well as the cross-population extended haplotype homozygosity (xp-EHH), were calculated in 50 kb sliding windows (25 kb slide). Chromosome boundaries are highlighted by an alternating light and dark grey pattern and chromosomes are numbered below.

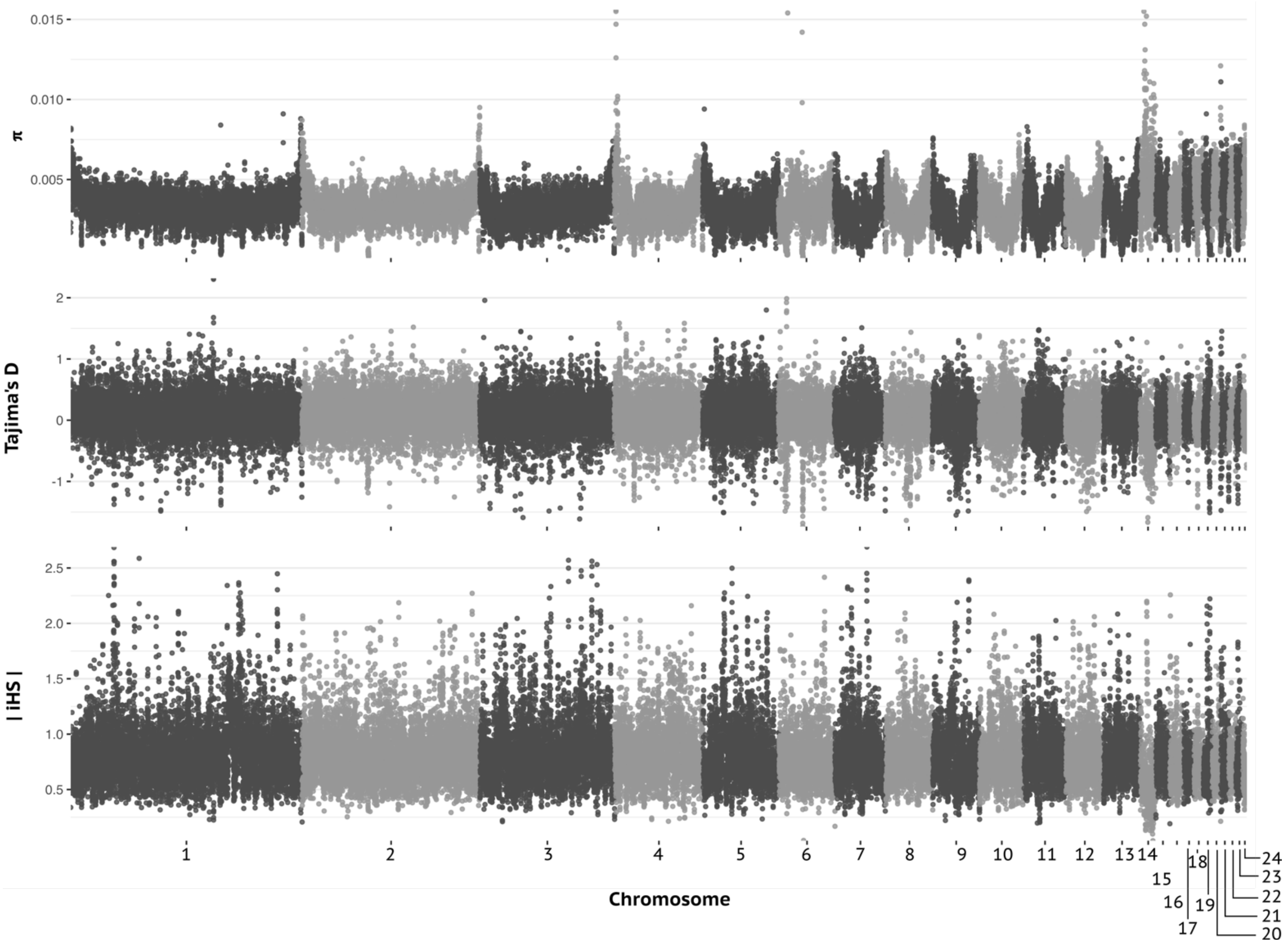

Figure S2: Autosomal distribution of SNP-based measures of within-population genomic diversity and selection signals in the Atlantic puffin subspecies *Fratercula arctica naumanni*. Nucleotide diversity (π), Tajima's D and the integrated haplotype homozygosity score (iHS) were calculated in 50 kb sliding windows (25 kb slide). Chromosome boundaries are highlighted by an alternating light and dark grey pattern.

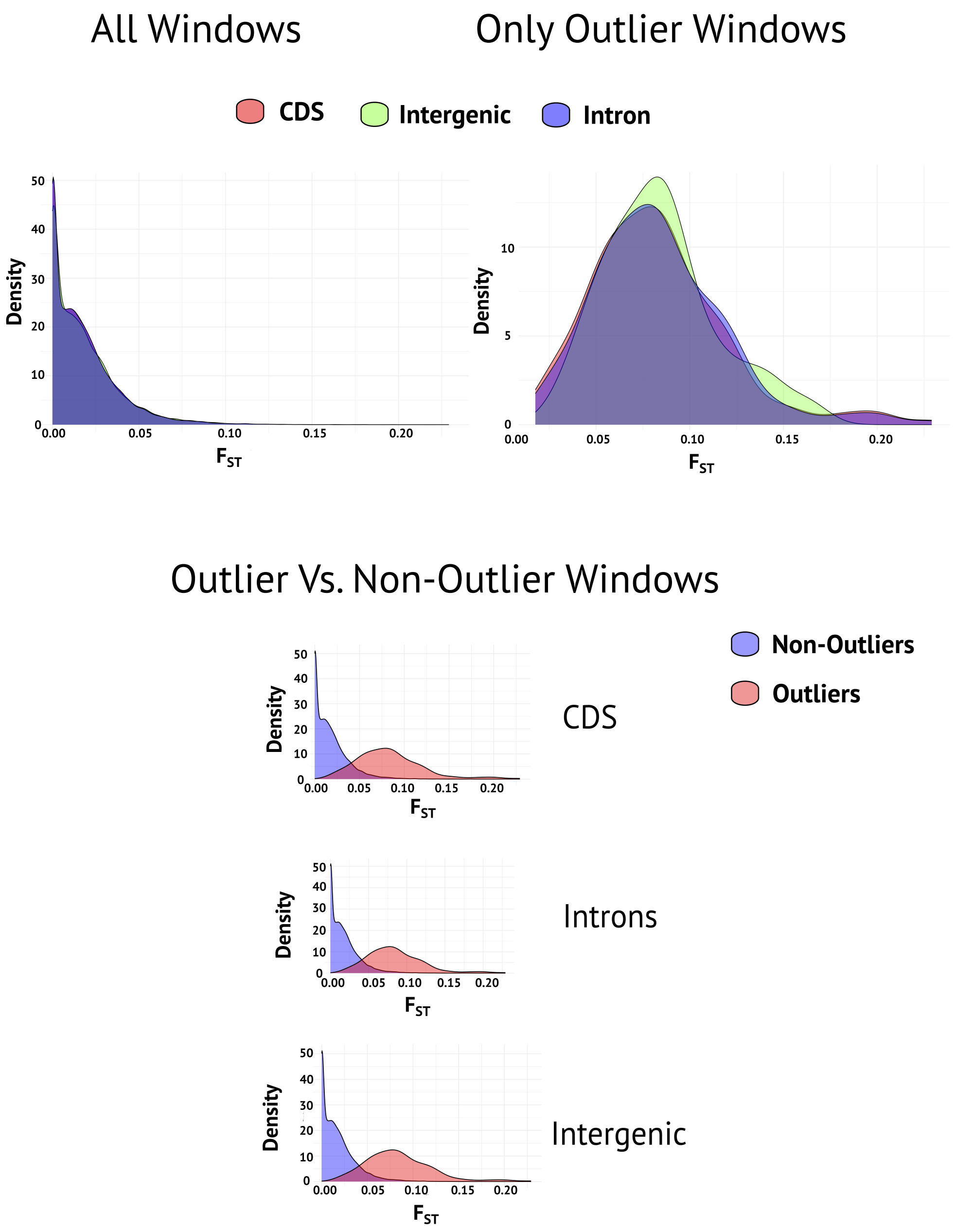

Figure S3: Distribution of relative differences in allele frequencies of 50 kb outlier and non-outlier windows compared between two Atlantic puffin (*Fratercula arctica*) subspecies - *F. a. naumanni* and *F. a. arctica*. The distributions are separated by A) genetic feature and B) outlier vs. non-outlier windows. FST was calculated across 43,638 50kb windows using whole genome-sequencing of 18 individuals. After calculating the de-correlated composite of multiple signals (DCMS) in each of the 43,638 windows, 255 outlier windows were found after testing for significance ($p < 0.05$) while accounting for the false discovery rate. Windows were designated to fall into genetic features if they were located within 8 kb.

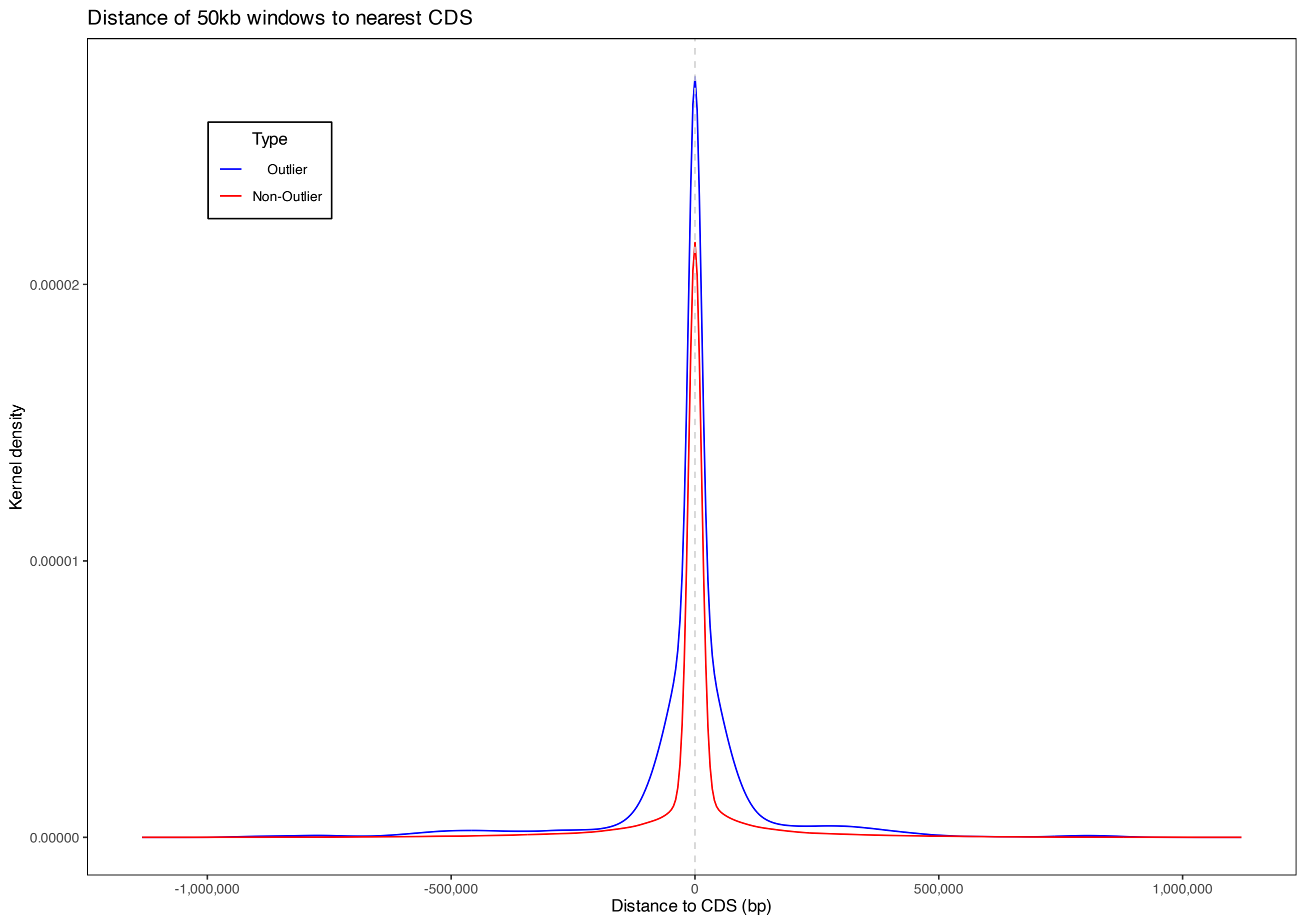

Figure S4: Distribution of the distance of 50 kb outlier and non-outlier windows to the nearest coding sequence in the Atlantic puffin (*Fratercula arctica*) genome comparing two subspecies (*F. a. arctica* and *F. a. naumanni*). The de-correlated composite of multiple signals (DCMS) was calculated across 43,638 50kb windows using whole genome-sequencing of 18 individuals. 255 outlier windows were found after testing for significance ($p < 0.05$) while accounting for the false discovery rate. CDS: coding sequence.

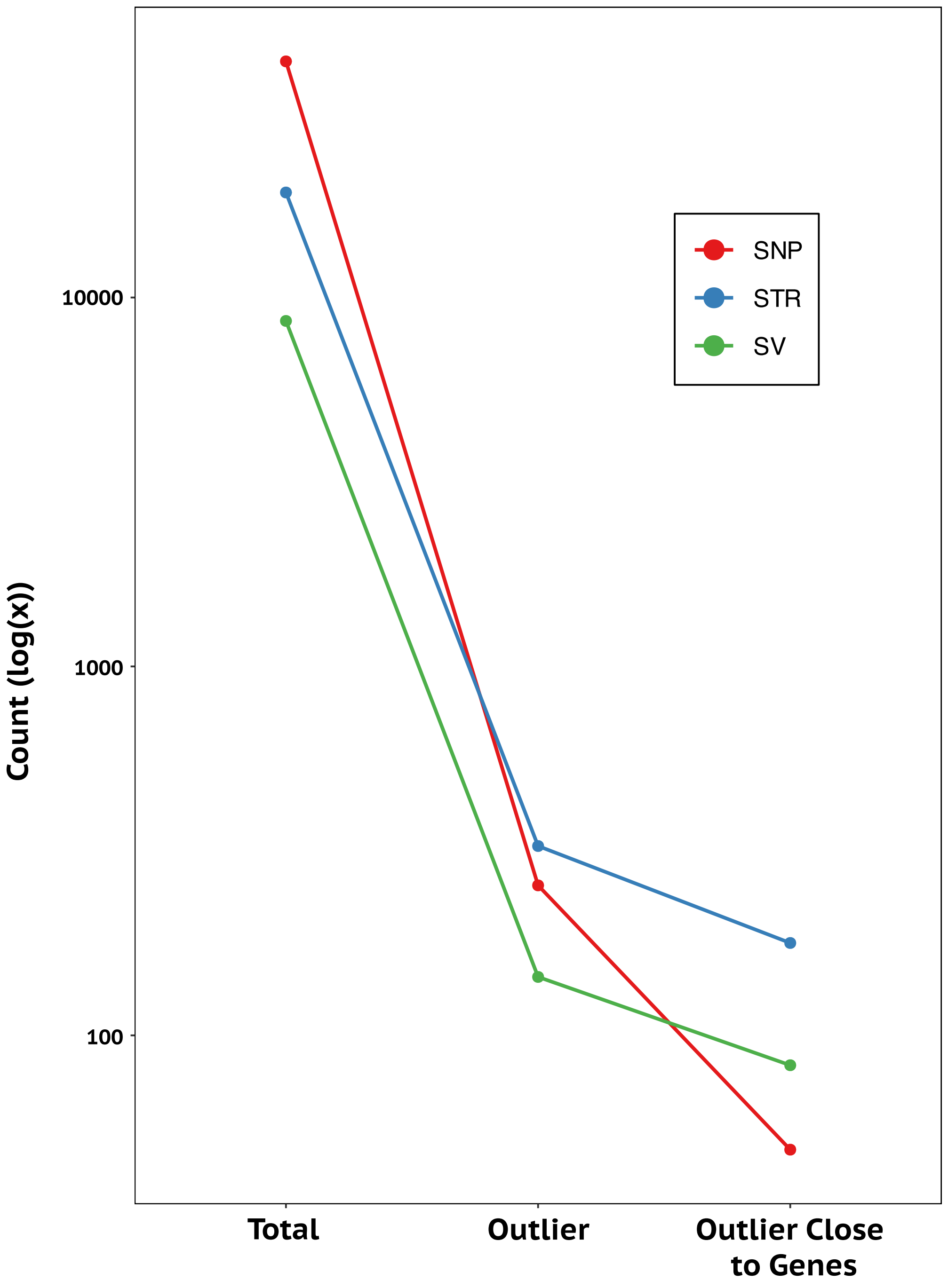

Figure S5: Contribution of outliers to the total amount of genetic variation across three different types of genetic variation (SNPs, STRs, SVs) between two Atlantic puffin subspecies. Outliers were identified by fitting F_{ST} or Jost's D values, arising from the comparison between *F. a. arctica* and *F. a. naumanni* genomes, to a normal distribution and correcting for multiple testing (FDR<0.05). "Outliers Close to Genes" includes outliers within 8 kb up or downstream of genes.

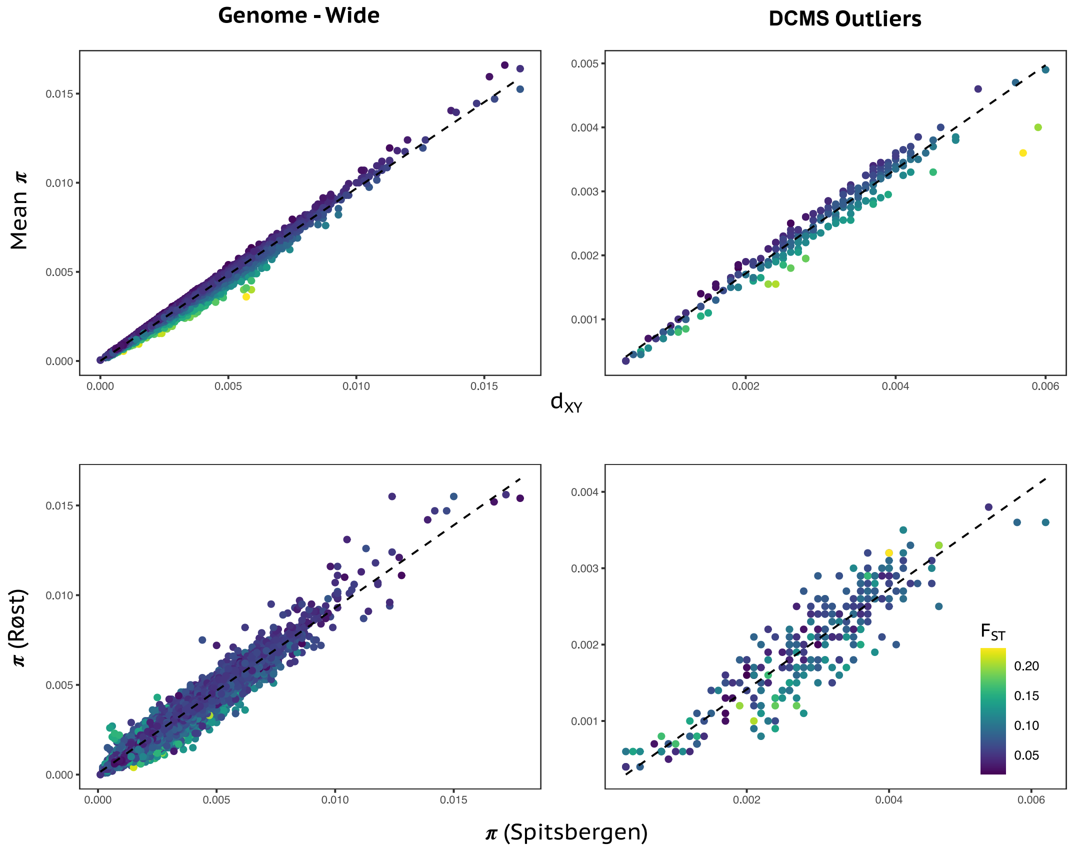

Figure S6: Correlations between relative (F_{ST}) and absolute (d_{XY}) genetic divergence, and nucleotide diversity (π) in autosomes and autosomal outliers between two Atlantic puffin subspecies. Values were calculated in 50 kb sliding windows (25 kb slide). The puffin colonies of Spitsbergen and Røst are representative of the subspecies *F. a. naumanni* and *F. a. arctica*, respectively. Outliers were detected with the de-correlated composite of multiple signals (DCMS). The DCMS combines five different SNP-based genome statistics commonly used for selection scans, which increases detection power. Significant outliers were set at q<0.05.

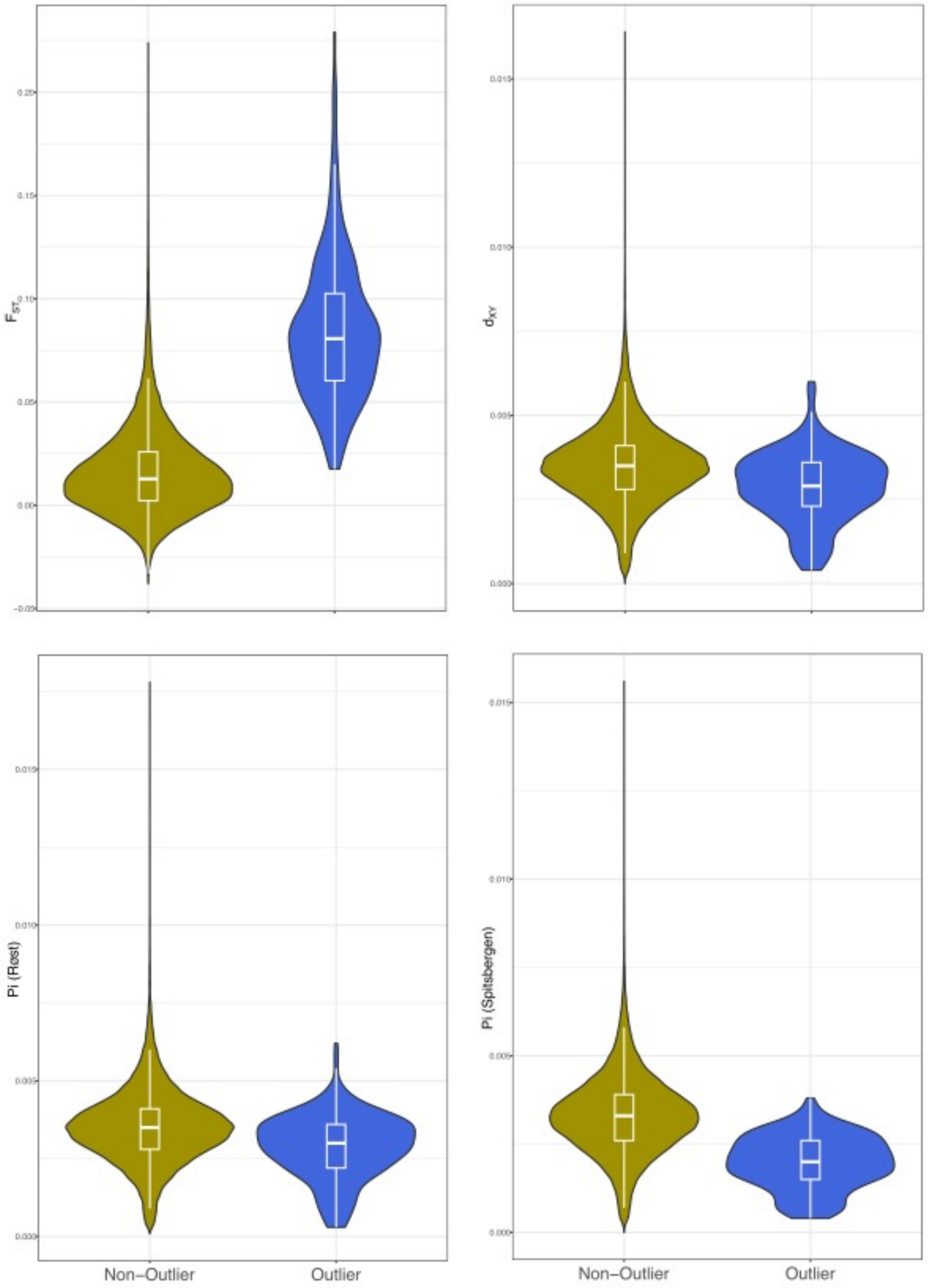

Figure S7: Relative (F_{ST}) and absolute (d_{XY}) genetic divergence, and nucleotide diversity (π) compared between outlier and non-outlier windows detected between two Atlantic puffin subspecies. Values were calculated in 50 kb sliding windows (25 kb slide). The puffin colonies of Spitsbergen and Røst are representative of the subspecies *F. a. naumanni* and *F. a. arctica*, respectively, and F_{ST} and d_{XY} values were calculated by comparing the genomes of both subspecies. Outliers were detected with the de-correlated composite of multiple signals (DCMS). The DCMS combines five different SNP-based genome statistics commonly used for selection scans, which increases detection power. Significant outliers were set at $q<0.05$.

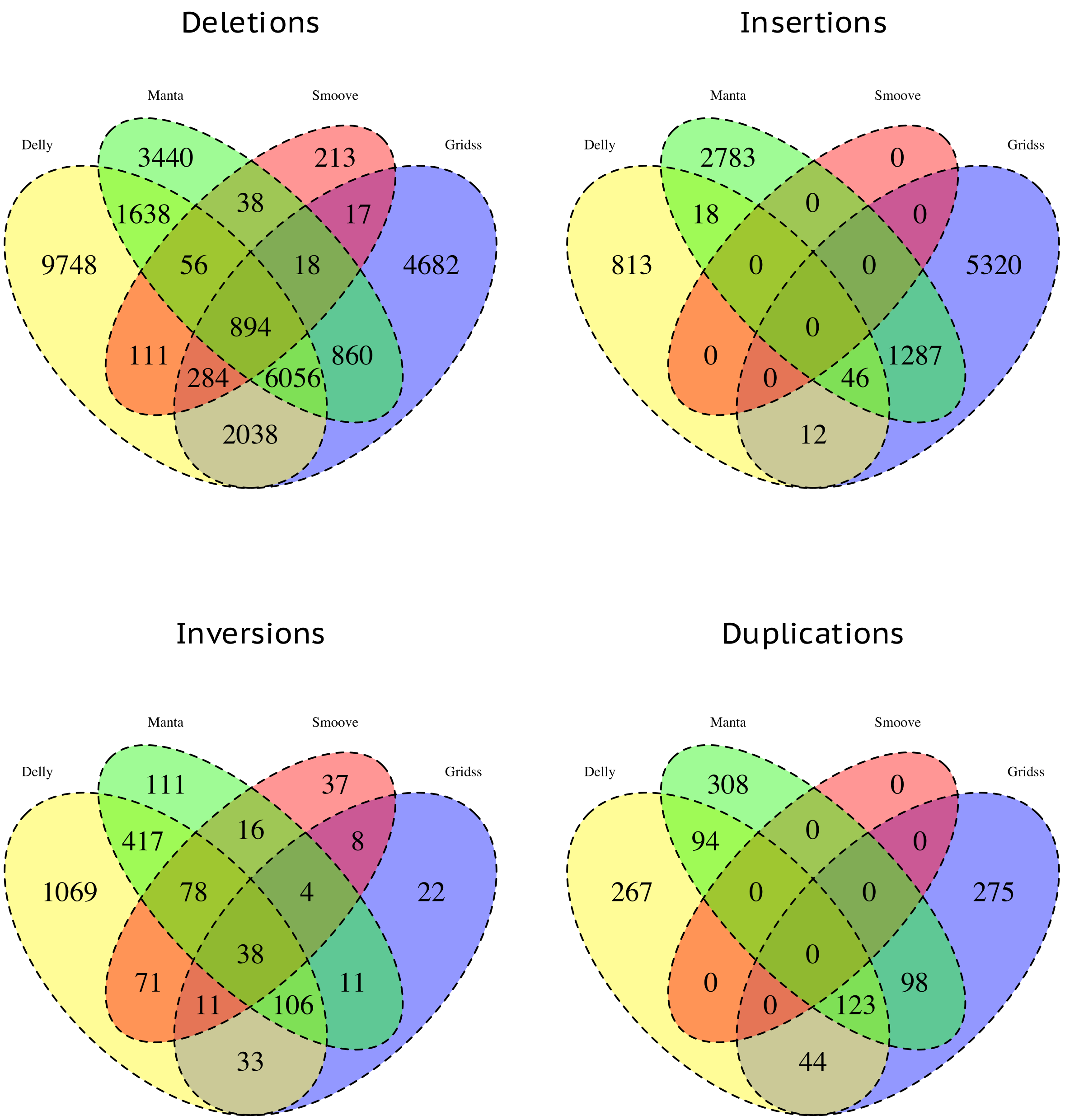

Figure S8: Overlap in detection of different types of autosomal structural variants (SVs) between four different programs. All four programs (Delly, Manta, Smoove, Gridss) were supplied with short-read sequencing data of 18 Atlantic puffin individuals sequenced to an average nuclear depth of coverage >20X.

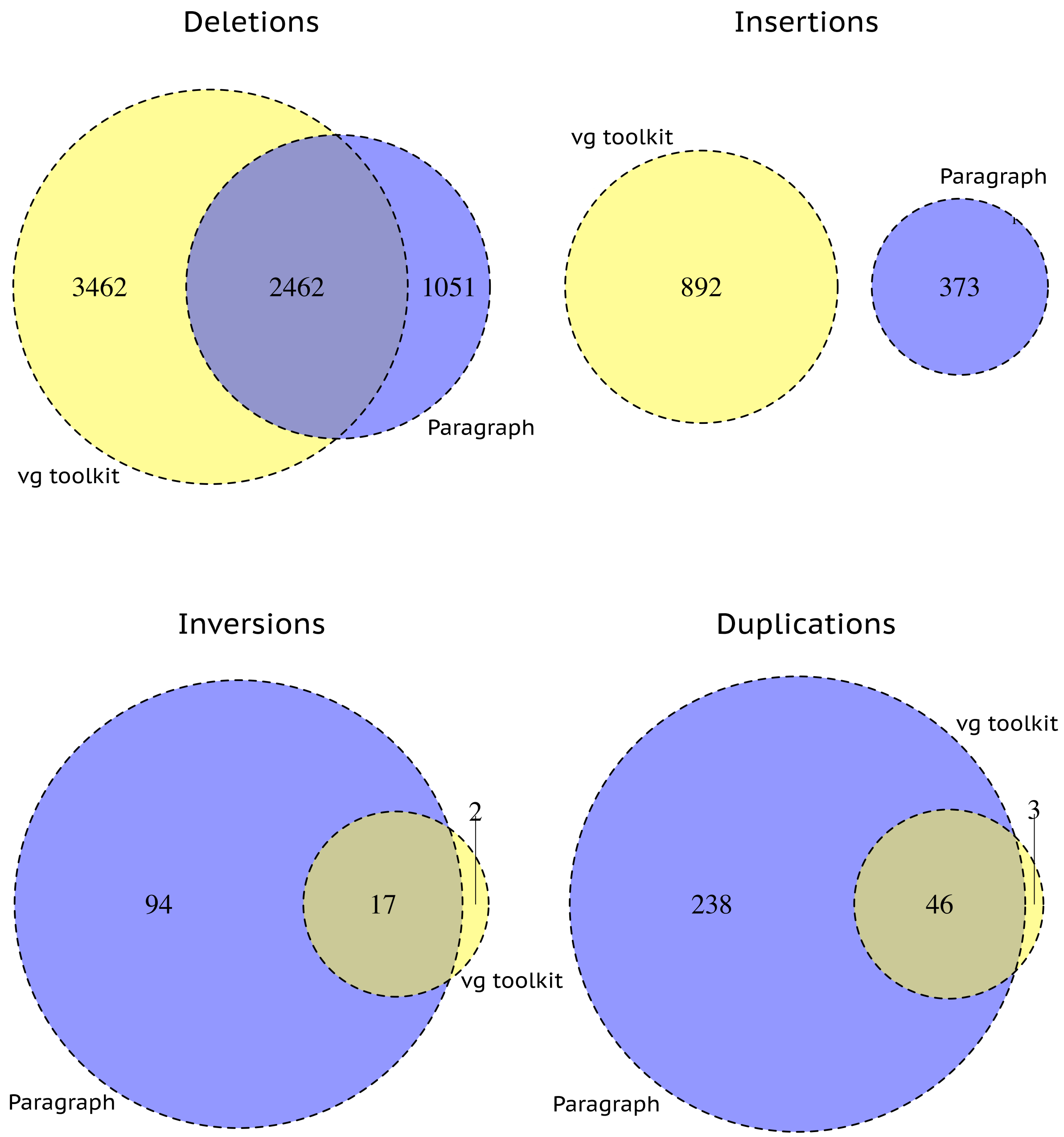

Figure S9: Overlap in genotyping of different types of autosomal structural variants (SVs) between vg toolkit and Paragraph. Both programs were supplied with short-read sequencing data of 18 Atlantic puffin individuals sequenced to an average nuclear depth of coverage >20X and a filtered set of previously detected SVs (detected by at least 2 of 4 tools).

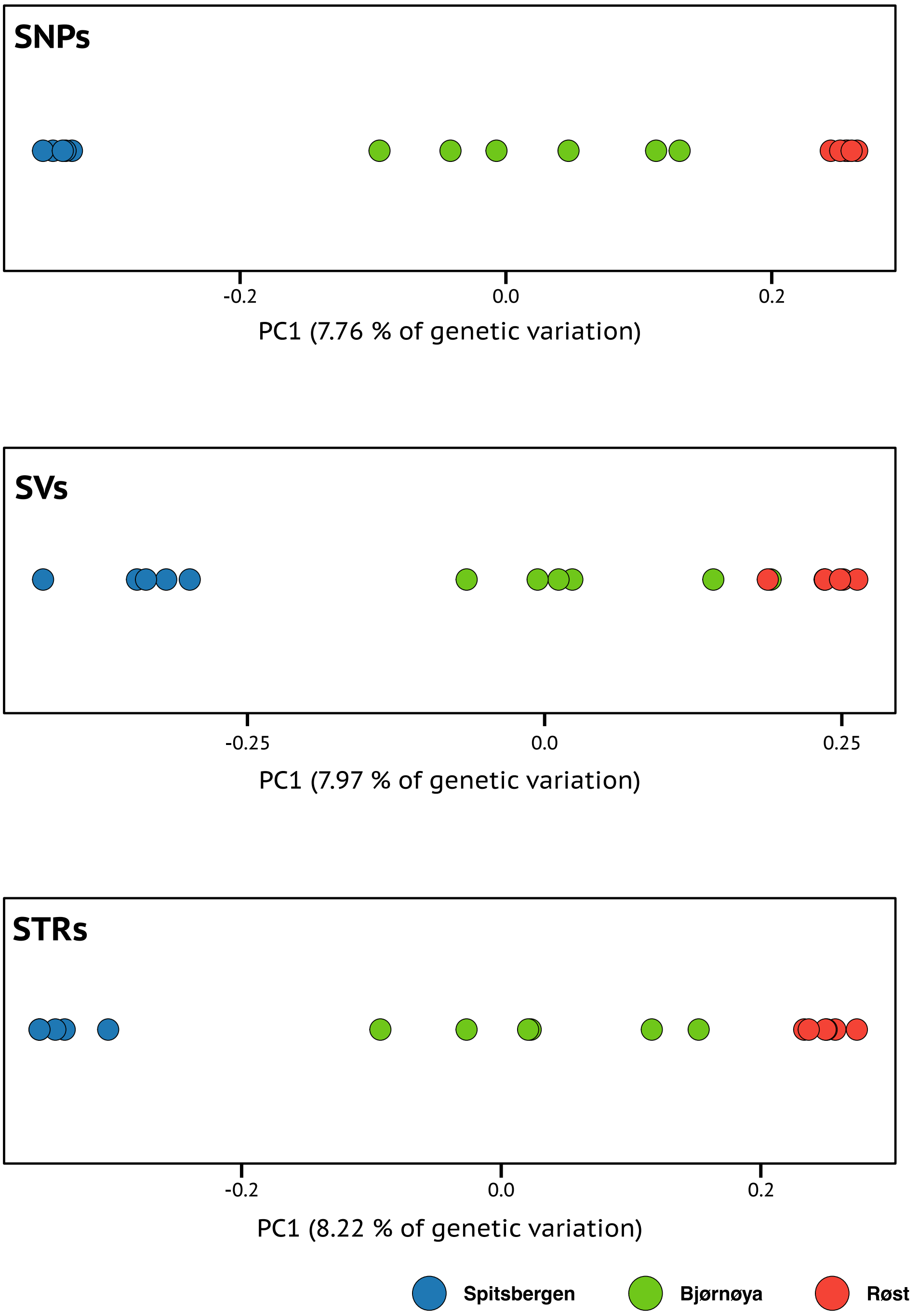

Figure S10: Autosomal population structure among three different Atlantic puffin colonies using three different types of genetic variation (SNPs, SVs, STRs). The principal component analyses were conducted using the program smartPCA. The SNP PCA was retrieved from Kersten et al. (2023). The puffin colonies of Spitsbergen and Røst are representative of the subspecies *F. a. naumanni* and *F. a. arctica*, respectively, while Bjørnøya is a hybrid population (Kersten et al. 2021, 2023).

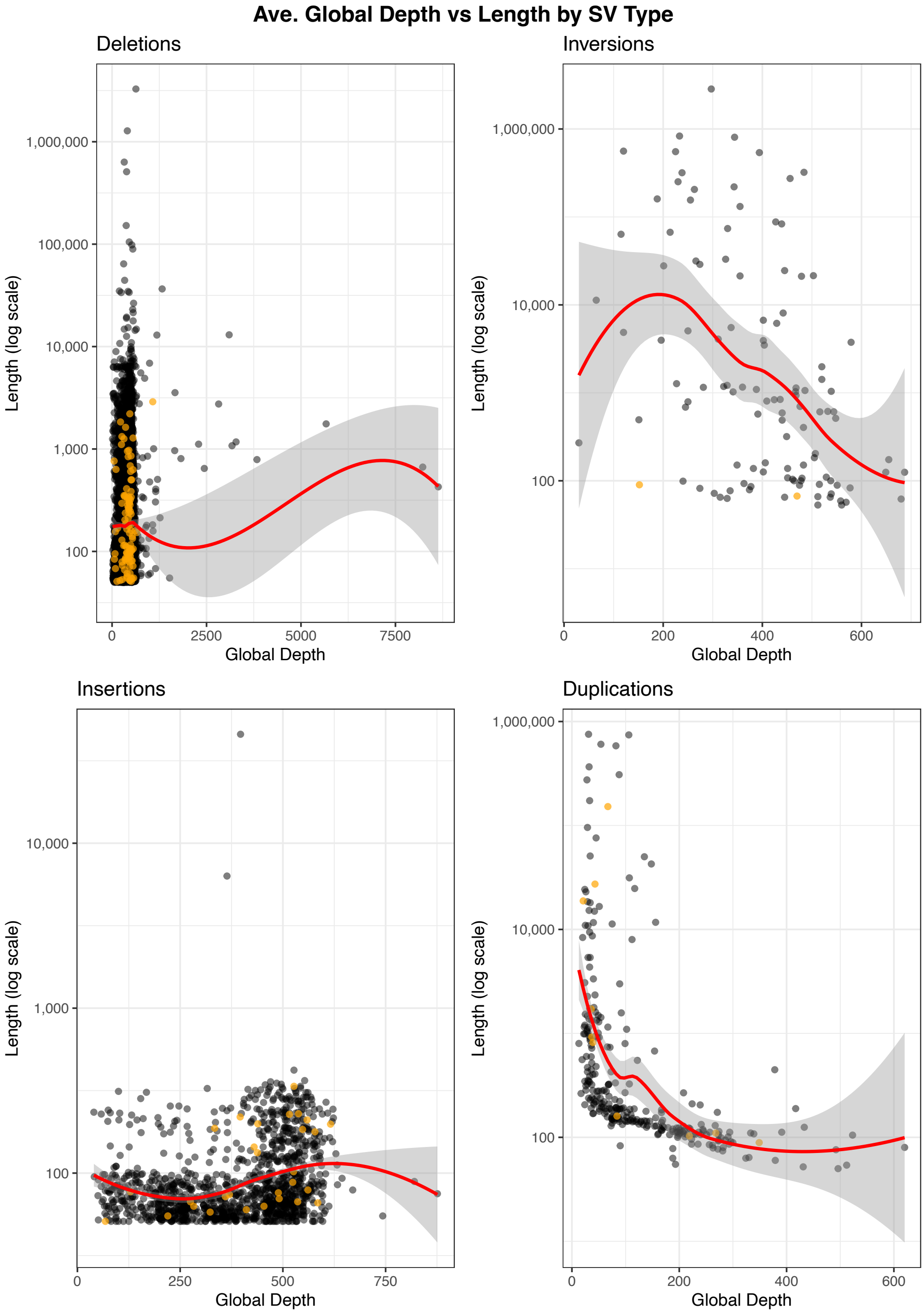

Figure S11: Length of detected structural variants (SV) as a function of global average sequencing depth using whole-genome sequencing of 18 Atlantic puffins (*Fratercula arctica*). The programs *Delly*, *Smoove*, *Manta*, and *Gridss* identified SVs, *Jasmine* merged the results of all four programs, and the *vg toolkit* and *Paragraph* were used to obtain genotype calls for the merged SVs. For each SV the average sequencing depth per individual was calculated and summed to obtain the "global average sequencing depth". The red line depicts a regression analysis with the grey shading highlighting the 95% confidence interval. Outlier SVs are highlighted in yellow.

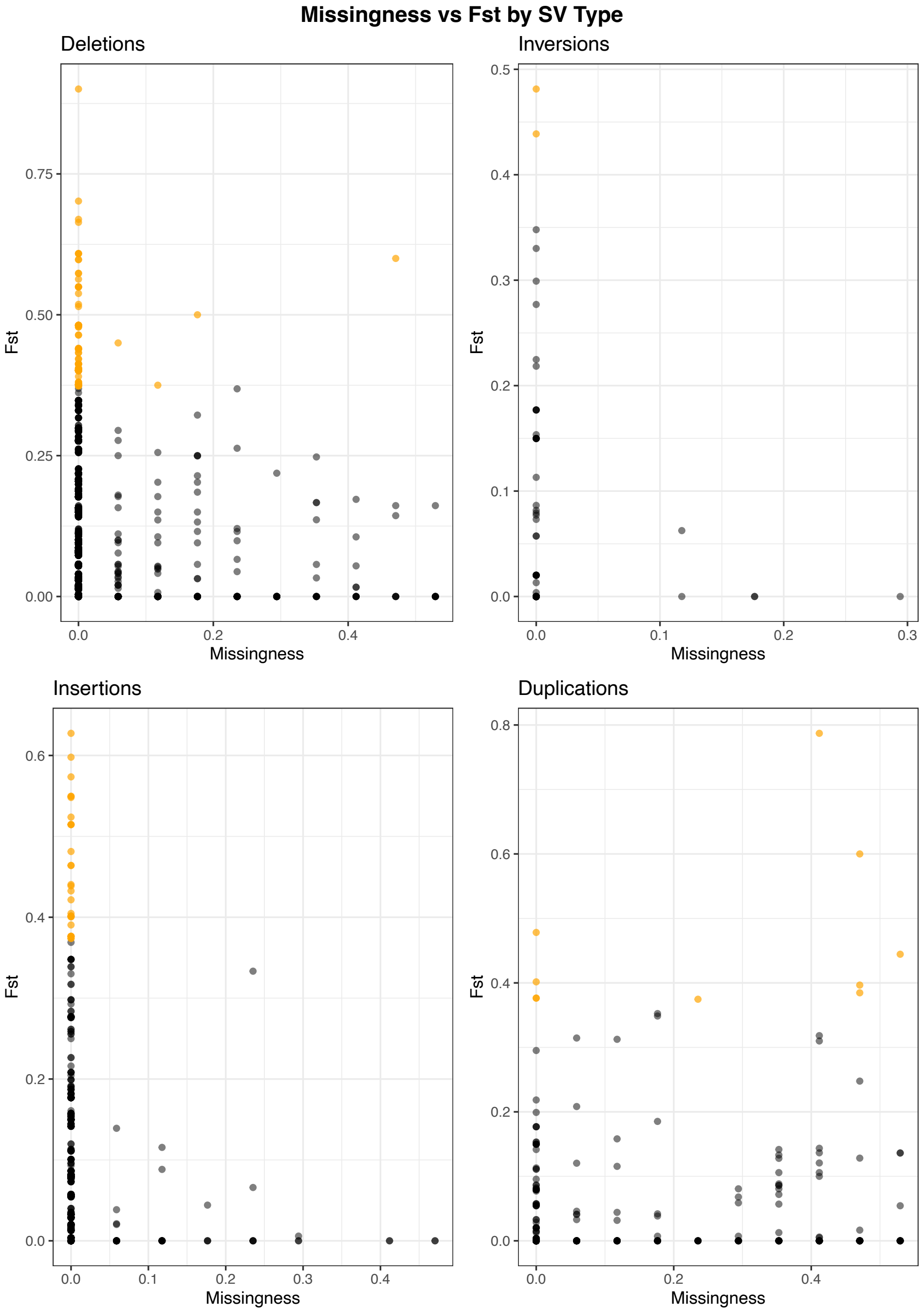

Figure S12: Relative differences in allele frequencies across structural variants (SVs) as a function of missingness using whole-genome sequencing of 18 Atlantic puffins (*Fratercula arctica*). The programs *Delly*, *Smoove*, *Manta*, and *Gridss* identified SVs, *Jasmine* merged the results of all four programs, and the *vg toolkit* and *Paragraph* were used to obtain genotype calls for the merged SVs. FST was calculated between six individuals of the subspecies *F. a. arctica* and six individuals of the subspecies *F. a. naumanni.* Missingness is the fraction of individuals without a specific genotyped SV. Outlier SVs are highlighted in yellow.

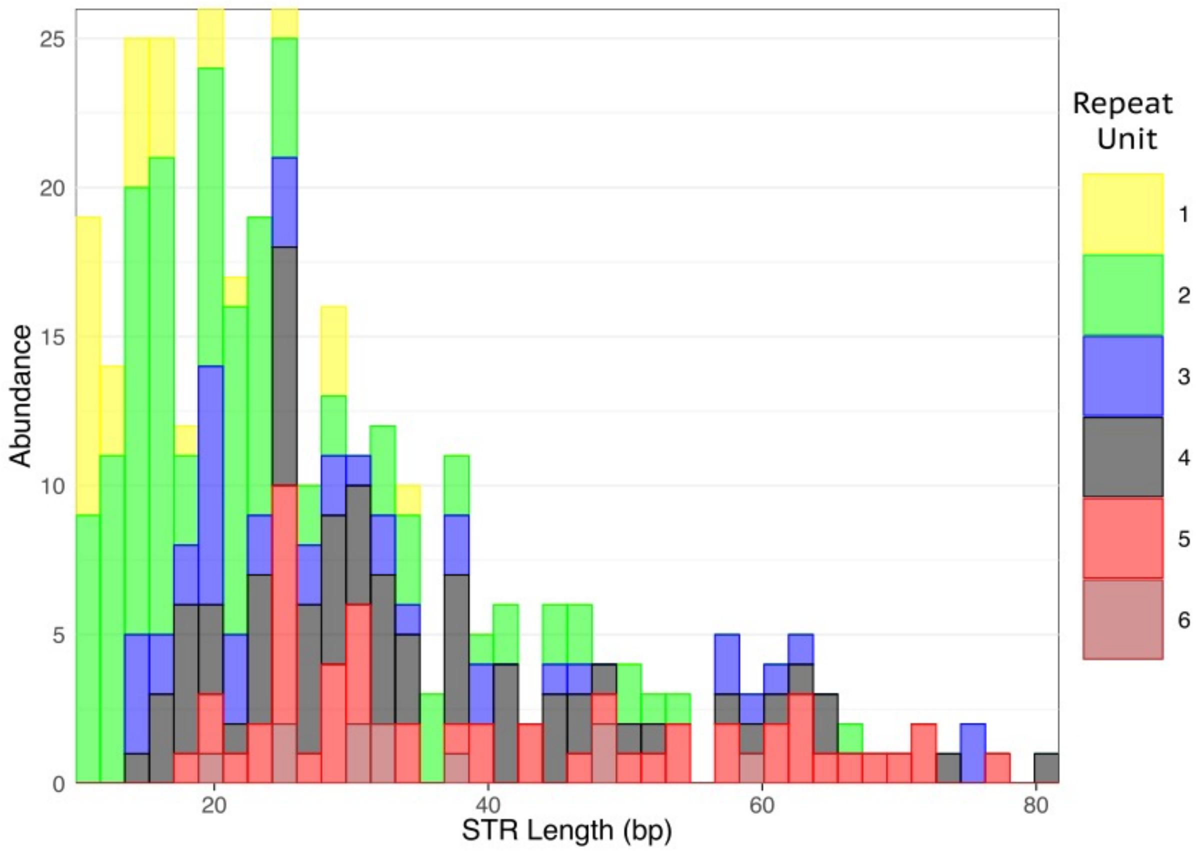

Figure S13: Length and abundance of outlier short tandem repeats (STRs) detected between two Atlantic puffin subspecies. Outlier STRs were identified by fitting Jost's D values of each STR to a normal distribution and correcting for multiple testing (FDR<0.05). Repeat unit denotes number of nucleotides within a repeating unit of an STR, i.e. a unit of 6 represents a hexamer.

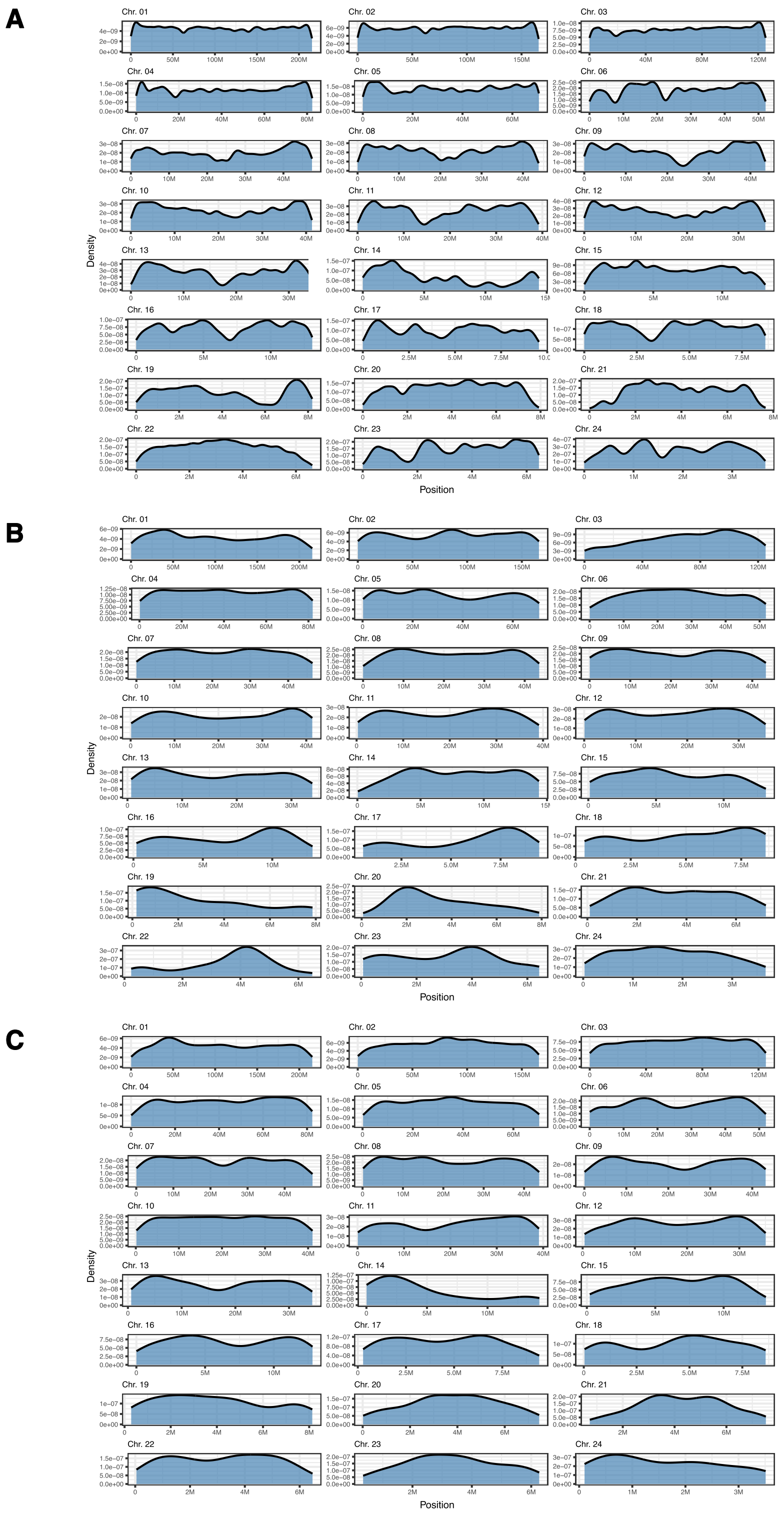

Figure S14: Abundance and location of genetic variation along the chromosomes of 18 Atlantic Puffins (*Fratercula arctica*). Genetic variation is represented by A) Single Nucleotide Polymorphisms, B) Structural Variants and C) Short Tandem Repeats and was detected using short-read sequencing to an average nuclear depth of coverage >20X.
